# The effect of chloroquine dose and primaquine on *Plasmodium vivax* recurrence: a WorldWide Antimalarial Resistance Network systematic review and individual patient pooled meta-analysis

**DOI:** 10.1016/S1473-3099(18)30348-7

**Published:** 2018-09

**Authors:** Robert J Commons, Julie A Simpson, Kamala Thriemer, Georgina S Humphreys, Tesfay Abreha, Sisay G Alemu, Arletta Añez, Nicholas M Anstey, Ghulam R Awab, J Kevin Baird, Bridget E Barber, Isabelle Borghini-Fuhrer, Cindy S Chu, Umberto D'Alessandro, Prabin Dahal, André Daher, Peter J de Vries, Annette Erhart, Margarete S M Gomes, Lilia Gonzalez-Ceron, Matthew J Grigg, Aliehsan Heidari, Jimee Hwang, Piet A Kager, Tsige Ketema, Wasif A Khan, Marcus V G Lacerda, Toby Leslie, Benedikt Ley, Kartini Lidia, Wuelton M Monteiro, Francois Nosten, Dhelio B Pereira, Giao T Phan, Aung P Phyo, Mark Rowland, Kavitha Saravu, Carol H Sibley, André M Siqueira, Kasia Stepniewska, Inge Sutanto, Walter R J Taylor, Guy Thwaites, Binh Q Tran, Hien T Tran, Neena Valecha, José Luiz F Vieira, Sonam Wangchuk, Timothy William, Charles J Woodrow, Lina Zuluaga-Idarraga, Philippe J Guerin, Nicholas J White, Ric N Price

**Affiliations:** a Global Health Division, Menzies School of Health Research and Charles Darwin University, Darwin, NT, Australia; b WorldWide Antimalarial Resistance Network, Clinical module, Darwin, NT, Australia; c Department of Infectious Diseases, Royal Brisbane and Women's Hospital, Herston, QLD, Australia; d Centre for Epidemiology and Biostatistics, Melbourne School of Population and Global Health, The University of Melbourne, Melbourne, VIC, Australia; e WorldWide Antimalarial Resistance Network, Oxford, UK; f Centre for Tropical Medicine, Nuffield Department of Clinical Medicine, University of Oxford, Oxford, UK; g ICAP, Columbia University Mailman School of Public Health, Addis Ababa, Ethiopia; h Department of Biology, Addis Ababa University, Addis Ababa, Ethiopia; i College of Natural Sciences, Addis Ababa University, Addis Ababa, Ethiopia; j Armauer Hansen Research Institute, Addis Ababa, Ethiopia; k Departamento de Salud Pública, Universidad de Barcelona, Barcelona, Spain; l Organización Panamericana de Salud, Oficina de país Bolivia, La Paz, Bolivia; m Mahidol-Oxford Tropical Medicine Research Unit, Faculty of Tropical Medicine, Mahidol University, Bangkok, Thailand; n Nangarhar Medical Faculty, Nangarhar University, Jalalabad Afghanistan; o Eijkman-Oxford Clinical Research Unit, Jakarta, Indonesia; p Infectious Diseases Society Sabah-Menzies School of Health Research Clinical Research Unit, Kota Kinabalu, Sabah, Malaysia; q Medicines for Malaria Venture, Geneva, Switzerland; r Shoklo Malaria Research Unit, Mahidol-Oxford Tropical Medicine Research Unit, Faculty of Tropical Medicine, Mahidol University, Mae Sot, Thailand; s Unit of Malariology, Institute of Tropical Medicine, Antwerp, Belgium; t Medical Research Council Unit, Fajara, The Gambia; u Institute of Drug Technology (Farmanguinhos), Oswaldo Cruz Foundation, Rio de Janeiro, Brazil; v Vice-Presidency of Research and Reference Laboratories, Oswaldo Cruz Foundation, Rio de Janeiro, Brazil; w Liverpool School of Tropical Medicine, Liverpool, UK; x Department of Internal Medicine, Tergooi Hospital, Hilversum, Netherlands; y Global Health Institute, Faculty of Medicine and Health Sciences, University of Antwerp, Belgium; z Superintendência de Vigilância em Saúde do Estado do Amapá -SVS/AP, Macapá, Amapá, Brazil; aa Federal University of Amapá, Macapá, Amapá, Brazil; ab Regional Centre for Public Health Research, National Institute for Public Health, Tapachula, Chiapas, Mexico; ac Department of Medical Parasitology, School of Medicine, Alborz University of Medical Sciences, Karaj, Iran; ad US President's Malaria Initiative, Malaria Branch, US Centers for Disease Control and Prevention, Atlanta, GA, USA; ae Global Health Group, University of California San Francisco, San Francisco, CA, USA; af Centre for Infection and Immunity Amsterdam, Academic Medical Center, Amsterdam, Netherlands; ag Division of Infectious Diseases, Tropical Medicine and AIDS, Academic Medical Center, Amsterdam, Netherlands; ah Department of Biology, Jimma University, Jimma, Ethiopia; ai International Centre for Diarrheal Diseases and Research, Dhaka, Bangladesh; aj Fundação de Medicina Tropical Dr Heitor Vieira Dourado, Manaus, Brazil; ak Programa de Pós-graduação em Medicina Tropical, Universidade do Estado do Amazonas, Manaus, Brazil; al Fundação Oswaldo Cruz, Instituto Leônidas e Maria Deane (FIOCRUZ-Amazonas), Manaus, Brazil; am Department of Infectious and Tropical Diseases, London School of Hygiene and Tropical Medicine, London, UK; an HealthNet-TPO, Kabul, Afghanistan; ao Department of Pharmacology and Therapy, Faculty of Medicine, Nusa Cendana University, Kupang, Indonesia; ap Centro de Pesquisa em Medicina Tropical de Rondônia, Porto Velho, Rondônia, Brazil; aq Universidade Federal de Rondônia, Porto Velho, Rondônia, Brazil; ar Tropical Diseases Clinical Research Center, Cho Ray Hospital, Ho Chi Minh City, Vietnam; as Department of Medicine, Kasturba Medical College, Manipal Academy of Higher Education, Manipal, Karnataka, India; at Manipal McGill Center for Infectious Diseases, Manipal Academy of Higher Education, Manipal, Karnataka, India; au Department of Genome Sciences, University of Washington, Seattle, WA, USA; av Instituto Nacional de Infectologia Evandro Chagas, Fundação Oswaldo Cruz, Rio de Janeiro, Brazil; aw Department of Parasitology, Faculty of Medicine, University of Indonesia, Jakarta, Indonesia; ax Oxford University Clinical Research Unit, Ho Chi Minh City, Vietnam; ay Malaria Research Centre, Delhi, India; az Federal University of Pará, Belém, Pará, Brazil; ba Public Health Laboratory, Department of Public Health, Ministry of Health, Thimphu, Bhutan; bb Infectious Diseases Unit, Clinical Research Centre, Queen Elizabeth Hospital, Kota Kinabalu, Sabah, Malaysia; bc Division of Clinical Sciences, St George's, University of London, London, UK; bd Grupo Malaria, Facultad de Medicina, Universidad de Antioquia, Medellín, Colombia

## Abstract

**Background:**

Chloroquine remains the mainstay of treatment for *Plasmodium vivax* malaria despite increasing reports of treatment failure. We did a systematic review and meta-analysis to investigate the effect of chloroquine dose and the addition of primaquine on the risk of recurrent vivax malaria across different settings.

**Methods:**

A systematic review done in MEDLINE, Web of Science, Embase, and Cochrane Database of Systematic Reviews identified *P vivax* clinical trials published between Jan 1, 2000, and March 22, 2017. Principal investigators were invited to share individual patient data, which were pooled using standardised methods. Cox regression analyses with random effects for study site were used to investigate the roles of chloroquine dose and primaquine use on rate of recurrence between day 7 and day 42 (primary outcome). The review protocol is registered in PROSPERO, number CRD42016053310.

**Findings:**

Of 134 identified chloroquine studies, 37 studies (from 17 countries) and 5240 patients were included. 2990 patients were treated with chloroquine alone, of whom 1041 (34·8%) received a dose below the target 25 mg/kg. The risk of recurrence was 32·4% (95% CI 29·8–35·1) by day 42. After controlling for confounders, a 5 mg/kg higher chloroquine dose reduced the rate of recurrence overall (adjusted hazard ratio [AHR] 0·82, 95% CI 0·69–0·97; p=0·021) and in children younger than 5 years (0·59, 0·41–0·86; p=0·0058). Adding primaquine reduced the risk of recurrence to 4·9% (95% CI 3·1–7·7) by day 42, which is lower than with chloroquine alone (AHR 0·10, 0·05–0·17; p<0·0001).

**Interpretation:**

Chloroquine is commonly under-dosed in the treatment of vivax malaria. Increasing the recommended dose to 30 mg/kg in children younger than 5 years could reduce substantially the risk of early recurrence when primaquine is not given. Radical cure with primaquine was highly effective in preventing early recurrence and may also improve blood schizontocidal efficacy against chloroquine-resistant *P vivax*.

**Funding:**

Wellcome Trust, Australian National Health and Medical Research Council, and Bill & Melinda Gates Foundation.

## Introduction

Chloroquine has been the mainstay of treatment for *Plasmodium vivax* for over 60 years.^1^, ^2^ The first observations of chloroquine-resistant *P vivax* were published in 1989,^3^, ^4^ and over the subsequent two decades several reports suggested that chloroquine-resistant *P vivax* was present in most vivax-endemic countries.^5^ Suboptimal treatment results in recurrent parasitaemia, from both recrudescent infections and relapses arising from reactivation of the dormant liver stages. Recurrent parasitaemia is associated with a cumulative risk of severe anaemia, increased mortality, and greater transmission potential.^6^, ^7^, ^8^

Treatment options for chloroquine-resistant *P vivax* include optimising chloroquine regimens or changing policy to a more effective blood schizontocidal agent. In countries where high-grade chloroquine-resistant *P vivax* is prevalent, national treatment guidelines have been revised to a universal policy of artemisinin combination therapy (ACT) for all species of malaria.^5^, ^9^ Where chloroquine remains the first-line treatment of *P vivax*, the treatment regimen can potentially be optimised, either by increasing the dose or duration of chloroquine, or by combining chloroquine with an additional drug with blood schizontocidal activity or the ability to reverse chloroquine resistance.^10^ Although early dose-finding studies showed excellent efficacy against *P vivax* at doses below 25 mg/kg, higher doses are well tolerated and might provide increased efficacy.^2^, ^11^ Alternatively, the addition of a hypnozoitocidal agent such as primaquine to chloroquine improves blood schizontocidal efficacy and reduces relapse.^12^, ^13^

**Research in context**

**Evidence before this study**

Using the search terms “vivax” and “chloroquine”, MEDLINE, Web of Science, Embase, and the Cochrane Database of Systematic Reviews were searched for articles published before Nov 29, 2017, that assessed the efficacy of chloroquine, with or without primaquine, for uncomplicated *Plasmodium vivax* malaria. A systematic review and meta-analysis showed that there was evidence of reduced chloroquine efficacy for *P vivax* present in most *P vivax* endemic countries. No reviews or pooled analyses had assessed the effect of chloroquine dose on the risk of recurrence.

**Added value of this study**

Our pooled analysis of individual patient data from 37 studies across 17 countries is, to our knowledge, the largest individual pooled analysis of *P vivax* clinical trials so far. Our findings highlight the substantial benefit of increasing the dose of chloroquine in children younger than 5 years and the additional benefit of adding primaquine to chloroquine.

**Implications of all the available evidence**

Chloroquine is currently under-dosed in children younger than 5 years. Increasing the target dose of chloroquine from 25 mg/kg to 30 mg/kg could significantly reduce the risk of *P vivax* recurrence within 42 days in children younger than 5 years who are not given primaquine. The risk of *P vivax* recurrence was reduced by an even greater degree by the addition of primaquine to chloroquine in all age groups, through prevention of relapse and probably improvement in blood schizontocidal efficacy. These measures warrant consideration by regional and global policy makers to reduce the risk of early *P vivax* recurrence.

To explore alternative strategies for improving chloroquine efficacy, we did a pooled analysis of individual patient data from prospective *P vivax* clinical trials to investigate the effect of chloroquine dose and primaquine co-administration on the risks of *P vivax* recurrence between day 7 and day 42.

## Methods

### Search strategy and selection criteria

We searched MEDLINE, Web of Science, Embase, and Cochrane Database of Systematic Reviews, according to the Preferred Reporting Items for Systematic Reviews and Meta-Analyses statement (appendix pp 2–5). Prospective therapeutic efficacy trials of uncomplicated *P vivax* malaria published in any language between Jan 1, 1960, and March 22, 2017, were identified using the following search terms: (malaria OR plasmodium) AND (amodiaquine OR atovaquone OR artemisinin OR arteether OR artesunate OR artemether OR artemotil OR azithromycin OR artekin OR chloroquine OR chlorproguanil OR cycloguanil OR clindamycin OR coartem OR dapsone OR dihydroartemisinin OR duo-cotecxin OR doxycycline OR halofantrine OR lumefantrine OR lariam OR malarone OR mefloquine OR naphthoquine OR naphthoquinone OR piperaquine OR primaquine OR proguanil OR pyrimethamine OR pyronaridine OR quinidine OR quinine OR riamet OR sulphadoxine OR tetracycline OR tafenoquine). Further details are provided in the appendix (p 6).^14^

The review process was done by two independent investigators (RJC and RNP), who also extracted the data. Disagreement was resolved through discussion. To ensure results were relevant to the current clinical landscape, only studies published after 2000 were included. Principal investigators were contacted and invited to share individual patient data and any additional unpublished data.

Studies assessing patients with *P vivax* monoinfection treated with chloroquine alone or chloroquine plus primaquine during the first 28 days after treatment were included. Individual patient data were uploaded into the WorldWide Antimalarial Resistance Network (WWARN) secure repository, anonymised, and processed according to a data management plan.^15^

All data included in this analysis were obtained in accordance with ethical approvals from the country of origin. The data are fully anonymised and cannot be traced back to identifiable individuals; these do not require review from an ethics committee according to the guidelines of the Oxford Central University Research Ethics Committee.

### Procedures

Chloroquine and primaquine doses were calculated from the number of tablets given to each patient. If these data were unavailable, doses were calculated based on the study protocol and assuming complete adherence. Individual patient records were excluded if the course of chloroquine was incomplete, the course of chloroquine or primaquine was intermittent, or if information on dose, parasitaemia, age, or weight were unavailable. Early primaquine was defined as the first dose of primaquine administered in the first 3 days of treatment (ie, before day 3).

Parasite prevalence at each study site was categorised as low (*P vivax* parasite rate <1·5%), moderate (≥1·5% and <4·0%), or high (≥4·0%) based on transmission estimates obtained from the Malaria Atlas Project and observed study site reinfection rates (appendix pp 16–17).^16^ Study sites were also categorised as having long or short *P vivax* relapse periodicity according to geographical location.^17^ Regions with short relapse periodicity were defined as having a median time to patent relapse of 47 days or fewer.^17^

### Outcomes

The primary outcome was the risk of *P vivax* recurrence between day 7 and day 42. Secondary outcomes were the risk of recurrence between day 7 and day 28 and early parasitological clearance, defined as the prevalence of parasitaemia on days 1, 2, and 3.^15^

### Statistical analysis

The risk of recurrence was calculated using Kaplan-Meier survival analyses. Patients were right censored at the day of their first recurrence, the day they were last seen, the day before a more than 18-day blood smear gap, or day 42, depending on which came first.^15^

Cox's proportional hazards regression was used to estimate the association between chloroquine dose and co-administration of primaquine with the rate of recurrence, adjusting for the potential confounders of age, sex, baseline parasitaemia, and regional relapse periodicity, and applying shared frailty for study sites to account for additional variation related to different sites. A linear association between chloroquine dose and the log rate of recurrence was checked visually, and the proportional hazards assumption tested using Schoenfeld residuals. If non-proportional hazards were present, interactions between terms and time were assessed. Owing to collinearity with relapse periodicity, geographical region and parasite prevalence were not included. Age was categorised into three groups (<5 years, 5 to <15 years, and ≥15 years) when a linear association with outcome was not present. Figures of risk of recurrence were estimated according to chloroquine dose and primaquine co-administration, adjusted for other confounders and assuming no study site effect.

The associations between chloroquine dose and microscopy-detectable parasite positivity in patients treated with chloroquine alone were analysed by logistic regression, with study sites included as a random effect. The association between the first day of parasite clearance and parasitaemia recurrence between day 7 and day 28 was assessed by Cox's proportional hazards regression.

Heterogeneity of studies was assessed by removal of one study site at a time and calculation of the coefficient of variation around parameter estimates. Additionally, baseline characteristics of included studies were compared with targeted studies that were not included.

Statistical analyses were done in Stata (version 15.0) and R (version 3.4.0), according to an a-priori statistical analysis plan.^18^ The review protocol is registered in PROSPERO, number CRD42016053310.

### Role of the funding source

The funders of the study had no role in study design, data collection, data analysis, data interpretation, or writing of the report. RNP had full access to all the data in the study and had final responsibility for the decision to submit for publication.

## Results

**Figure 1:**
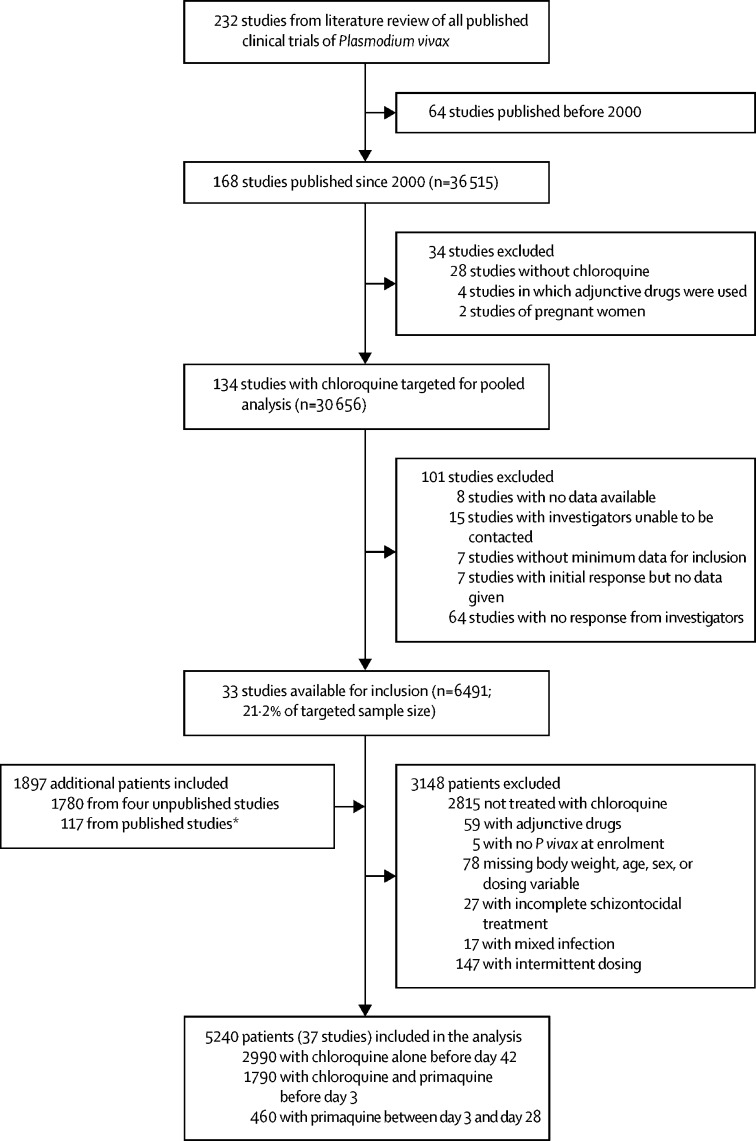
Study profile *Additional patient data available from published studies that were not described in the published enrolment cohorts.

232 published *P vivax* clinical trials were identified, 134 of which included patients treated with chloroquine and were published between Jan 1, 2000, and March 22, 2017 (figure 1). Individual patient data were available from 33 published studies^19^, ^20^, ^21^, ^22^, ^23^, ^24^, ^25^, ^26^, ^27^, ^28^, ^29^, ^30^, ^31^, ^32^, ^33^, ^34^, ^35^, ^36^, ^37^, ^38^, ^39^, ^40^, ^41^, ^42^, ^43^, ^44^, ^45^, ^46^, ^47^, ^48^, ^49^, ^50^, ^51^ including 6491 patients (21·2%) of the target sample size of 30 656. Additionally, patient data from four unpublished studies including 1780 patients and an additional 117 patients related to patient cohorts from the published studies, but not described in the manuscripts, were available (figure 1; appendix pp 7–9, 13–15). 5573 (66·4%) of 8388 patients from these studies were treated with chloroquine, of whom 333 (6·0%) were excluded because of protocol violations. Of the 5240 patients included in the analysis, 2990 (57·1%) were treated with chloroquine alone, 1790 (34·2%) were treated with chloroquine plus early primaquine commencing before day 3, and 460 (8·8%) received primaquine after day 3. Patients were followed up for 28 days in 20 studies (n=3041), 29–42 days in seven studies (n=675), 43–63 days in four studies (n=583), and more than 63 days in six studies (n=941). Mg/kg dosing was calculated from the number of tablets given for 3197 (61·0%) of 5240 patients, with the remainder extrapolated from the protocol, assuming complete adherence.

The median age of patients was 20 years (IQR 10–31; range 3 months to 88 years), with 450 (8·6%) aged younger than 5 years (table 1). 3773 (72·0%) patients were from the Asia-Pacific region, compared with 776 (14·8%) from the Americas and 691 (13·2%) from Africa. 2187 (41·7%) patients came from areas of short relapse periodicity. Patients treated with chloroquine alone were younger and more likely to come from regions of long relapse periodicity, have higher baseline parasitaemia, present with anaemia (haemoglobin <10 g/dL), and come from Africa rather than the Americas (table 1). The corresponding proportions of patients from studies targeted for inclusion, but not included in the pooled analysis, were similar (appendix pp 18–21).

**Table 1:** Demographics and baseline characteristics

	**Chloroquine alone (n=2990)**	**Chloroquine and early primaquine (n=1790)**	Overall (n=5240)*
**Sex**			
Female	1104 (36·9%)	571 (31·9%)	1786 (34·1%)
Male	1886 (63·1%)	1219 (68·1%)	3454 (65·9%)
**Age (years)**			
Median (IQR)	17·0 (8·0–28·0)	23·5 (13·0–36·0)	20·0 (10·0–31·0)
<5	359 (12·0%)	88 (4·9%)	450 (8·6%)
5 to <15	916 (30·6%)	413 (23·1%)	1403 (26·8%)
≥15	1715 (57·4%)	1289 (72·0%)	3387 (64·6%)
**Weight (kg)**			
Median (IQR)	45·0 (20·0–56·0)	51·0 (36·0–62·0)	48·0 (25·0–58·0)
5 to <15	342 (11·4%)	101 (5·6%)	445 (8·5%)
15 to <25	574 (19·2%)	208 (11·6%)	813 (15·5%)
25 to <35	235 (7·9%)	120 (6·7%)	388 (7·4%)
35 to <45	320 (10·7%)	185 (10·3%)	564 (10·8%)
45 to <55	649 (21·7%)	413 (23·1%)	1250 (23·9%)
55 to <80	777 (26·0%)	656 (36·6%)	1577 (30·1%)
≥80	93 (3·1%)	107 (6·0%)	203 (3·9%)
**Relapse periodicity**			
Long	1914 (64·0%)	902 (50·4%)	3053 (58·3%)
Short	1076 (36·0%)	888 (49·6%)	2187 (41·7%)
**Geographical region**			
Asia-Pacific	2112 (70·6%)	1203 (67·2%)	3773 (72·0%)
The Americas	289 (9·7%)	487 (27·2%)	776 (14·8%)
Africa	589 (19·7%)	100 (5·6%)	691 (13·2%)
**Prevalence of *Plasmodium vivax***			
Low	1243 (41·6%)	195 (10·9%)	1459 (27·8%)
Moderate	607 (20·3%)	744 (41·6%)	1723 (32·9%)
High	1140 (38·1%)	851 (47·5%)	2058 (39·3%)
**Enrolment clinical variables**			
Parasitaemia, parasites per μL	4000 (1480–8290)	3000 (1000–7520)	3809 (1380–8360)
Haemoglobin, g/dL†	12·2 (2·1)	12·7 (2·1)	12·4 (2·1)
Anaemia, haemoglobin <10 g/dL	263/1991 (13·2%)	138/1605 (8·6%)	428/3840 (11·1%)
Gametocytes present	1473/1642 (89·7%)	850/916 (92·8%)	2502/2763 (90·6%)
Fever, temperature >37·5°C	1280/2757 (46·4%)	687/1546 (44·4%)	2267/4752 (47·7%)

Data are number (%), median (IQR), mean (SD), or n/N (%). Some percentages do not add up to 100 because of rounding.

* Includes 460 patients treated with chloroquine and primaquine who started primaquine after the first 3 days;

† Data not available for 1400 of 5240 patients: 999 in the chloroquine alone group and 185 in the chloroquine and primaquine group.

In the 2990 patients from 26 studies treated with chloroquine alone, the median total dose of chloroquine was 25·4 mg/kg (IQR 24·2–28·1; range 6·8–75·0) with 1041 patients (34·8%) receiving less than the WHO recommended target dose of chloroquine of 25 mg/kg (appendix pp 10, 22). This proportion increased with weight, age, male sex, and regions of short relapse periodicity (appendix pp 11, 22). Under-dosing in children younger than 5 years was similar between those dosed with chloroquine syrup or a liquid mixture from crushed tablets (11 [17%] of 66) and those dosed with divided tablets (39 [13%] of 293; p=0·48).

Information on acute vomiting was available in eight studies (n=557), with 20 (4%) of 557 patients vomiting at least one dose of chloroquine within 60 min of administration. After adjusting for age, sex, baseline parasitaemia, presence of baseline fever, and relapse periodicity, chloroquine dose was not a significant risk factor for vomiting (adjusted odds ratio [AOR] 1·14, 95% CI 0·96–1·34; p=0·13; appendix p 24).

505 patients treated with chloroquine alone had recurrent parasitaemia between day 7 and day 42. In patients followed up for 42 days, 69 (23%) of 298 recurrences within this period occurred by day 28. The cumulative risk of recurrence was 10·4% (95% CI 9·3–11·6) at day 28 and 32·4% (29·8–35·1) at day 42. The risks of recurrence for individual studies are presented in the appendix (p 12).

**Figure 2:**
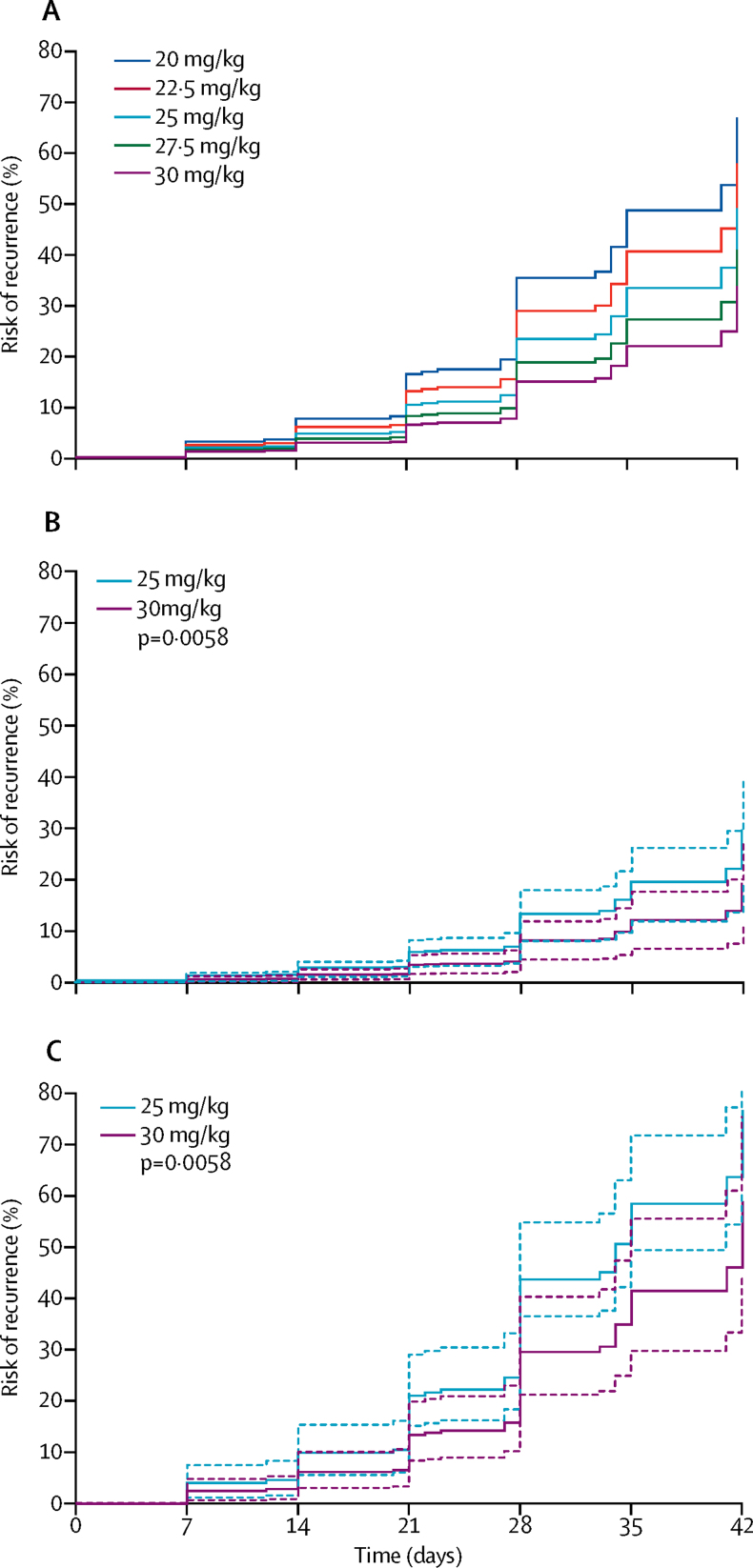
Risk of recurrence in patients younger than 5 years receiving chloroquine alone with (A) varied chloroquine doses, and in (B) long periodicity and (C) short periodicity regions Dashed lines are the 95% CIs. Adjusted for age, sex, and baseline parasitaemia. Assumes zero effect from study site. p values are derived from a Cox model.

After controlling for age, parasitaemia, regional relapse periodicity, and sex, a 5 mg/kg increase in chloroquine dose reduced the rate of recurrence between day 7 and day 42 (adjusted hazard ratio [AHR] for every 5 mg/kg dose increase 0·82, 95% CI 0·69–0·97; p=0·021; table 2). After stratifying the model by geographical region, the AHR for a 5 mg/kg increase in chloroquine dose was 0·75 (95% CI 0·59–0·96; p=0·022) in the Asia-Pacific, 0·84 (0·59–1·20; p=0·35) in the Americas, and 0·95 (0·72–1·25; p=0·72) in Africa. Age, baseline parasitaemia, and short regional relapse periodicity were also associated with parasite recurrence between day 7 and day 42 (table 2). The effect of dose was greatest in children aged younger than 5 years (AHR for every 5 mg/kg increase in dose 0·59, 95% CI 0·41–0·86; p=0·0058; figure 2; appendix p 25). Since the proportional hazards assumption did not hold for chloroquine dose in patients aged 5 years or older, variation of chloroquine dose hazard ratio with time was assessed. For these age groups (5 to <15 years and ≥15 years), the AHR of chloroquine dose varied with time, with chloroquine dose associated with a reduced rate of recurrence from day 22 to day 42 (5 to <15 years AHR 0·66, 95% CI 0·45–0·96; p=0·030 and ≥15 years 0·83, 0·61–1·15; p=0·27), but there was no reduction with dose up to day 21 (appendix p 25). Sensitivity analyses in which one study site was removed at a time revealed no apparent bias relating to individual study sites from included studies (appendix pp 33–36).

**Table 2:** Risk factors for *Plasmodium vivax* recurrence between day 7 and day 42 in patients treated with chloroquine alone

		**Number of patients**	**Number with recurrence by day 42**	**Univariable analyses**		Multivariable analyses*	
				Crude HR (95% CI)	p value	Adjusted HR (95% CI)	p value
Chloroquine dose, every 5 mg/kg increase		2990	505	0·95 (0·80–1·12)	0·53	0·82 (0·69–0·97)	0·021
Age, every 1-year increase		2990	505	0·97 (0·96–0·97)	<0·0001	0·96 (0·96–0·97)	<0·0001
Age category, years							
	≥15	1715	223	Reference	..	..	..
	<5	359	100	2·53 (1·94–3·30)	<0·0001	..	..
	5 to <15	916	182	1·89 (1·52–2·35)	<0·0001	..	..
Weight, every 5 kg increase		990	2505	0·90 (0·88–0·93)	<0·0001	..	..
Sex							
	Male	1886	316	Reference	..	Reference	..
	Female	1104	189	0·94 (0·78–1·13)	0·50	0·96 (0·80–1·16)	0·69
Enrolment clinical variables							
	Parasitaemia, parasites per μL every ten-times increase	2990	505	1·29 (1·10–1·53)	0·0023	1·27 (1·07–1·49)	0·0049
	Haemoglobin, every 1 g/dL increase	1991	352	0·87 (0·83–0·92)	<0·0001	..	..
	Anaemia, haemoglobin <10 g/dL	1991	352	1·75 (1·31–2·35)	0·0002	..	..
	Fever, temperature >37·5°C	2757	478	1·27 (1·05–1·53)	0·015	..	..
	Gametocytes present	1642	335	0·98 (0·67–1·44)	0·92	..	..
Relapse periodicity							
	Long	1914	144	Reference	..	Reference	..
	Short	1076	361	18·16 (7·47–44·19)	<0·0001	21·61 (8·69–53·76)	<0·0001
Region							
	Asia-Pacific	2112	374	Reference	..	..	..
	Africa	589	76	0·14 (0·04–0·53)	0·0037	..	..
	The Americas	289	55	0·20 (0·02–2·22)	0·19	..	..
Prevalence							
	Low	1243	131	Reference	..	..	..
	Moderate	607	55	0·98 (0·24–4·00)	0·98	..	..
	High	1140	319	2·21 (0·46–10·62)	0·32	..	..
Dose calculation method							
	Per protocol	1223	215	Reference	..	..	..
	Tablet counts	1767	290	1·26 (0·31–5·11)	0·75	..	..

Weight was excluded owing to collinearity with age. Region and *P vivax* prevalence were excluded owing to collinearity with regional periodicity. HR=hazard ratio.

* The assumption of proportional hazards held for the overall model (p=0·06 for global test), with a p value of 0·007 specifically for chloroquine dose.

Within 24 h of starting treatment, 1169 (56·5%) of 2070 patients had cleared their detectable parasitaemia, increasing to 2095 (80·9%) of 2590 on day 2 and 2369 (94·8%) of 2499 on day 3. Low chloroquine dose (<25 mg/kg) was a risk factor for parasitaemia on day 1 in the univariable analysis (odds ratio 2·09, 95% CI 1·24–3·51; p=0·0056), as were male sex, older age, and higher baseline parasitaemia (appendix p 26). After controlling for con-founding factors, the association between low chloroquine dose and parasitaemia on day 1 was attenuated (AOR 1·65, 95% CI 0·98–2·78; p=0·060; appendix p 26). There was no relationship between chloroquine dose and parasite clearance on day 2 (AOR 1·52, 95% CI 0·78–2·97; p=0·22) or day 3 (1·39, 0·60–3·22; p=0·44).

**Figure 3:**
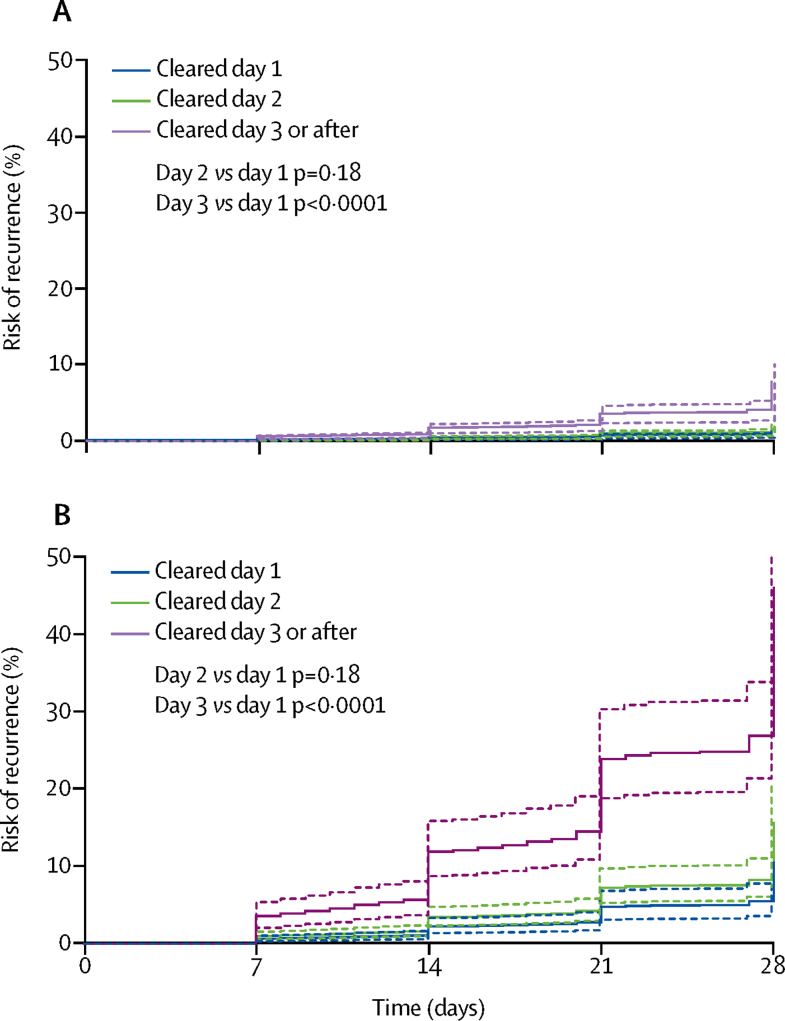
Risk of recurrence according to day of parasite clearance in patients receiving chloroquine alone in (A) long and (B) short periodicity regions Dashed lines are the 95% CIs. Adjusted for age, sex, baseline parasitaemia, and chloroquine dose. Assumes zero effect from study site. p values are derived from a Cox model.

In patients treated with chloroquine alone who were assessed at day 28, 32 (23%) of 139 who were parasitaemic on day 3 had recurrent *P vivax* between day 7 and day 28, compared with 229 (9%) of 2657 who had already cleared their parasitaemia (p<0·0001). After controlling for confounding factors, parasite clearance on or after day 3 was associated with an increased rate of recurrence between day 7 and day 28 compared with parasite clearance on day 1 (AHR 3·57, 95% CI 2·09–6·11; p<0·0001; appendix p 27). The higher rate of recurrence with delayed parasite clearance was more apparent in studies from short periodicity regions (figure 3).

In 17 studies, 1790 patients were treated with chloroquine and early primaquine. 917 (51·2%) patients from 11 of these studies had a target dose of primaquine between 3·5 mg/kg and less than 5·0 mg/kg, and 873 (48·8%) from six studies had a target dose of at least 5·0 mg/kg. Overall, patients were administered a median dose of primaquine of 4·7 mg/kg (IQR 3·4–6·7; range 0·3–13·1; appendix pp 10, 23). 1046 (58·4%) of 1790 primaquine regimens were 14 days long (range 7–14 days; appendix p 28).

**Figure 4:**
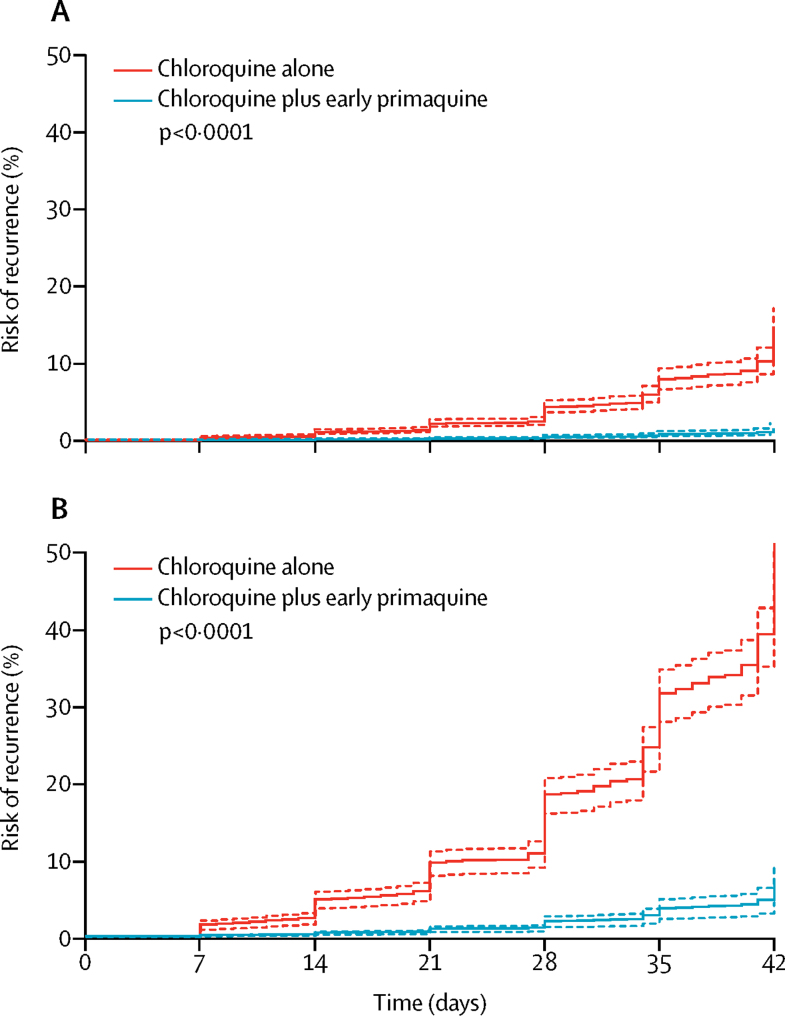
Risk of recurrence in patients receiving chloroquine alone or chloroquine plus early primaquine in (A) long and (B) short periodicity regions Dashed lines are the 95% CIs. Adjusted for age, sex, and baseline parasitaemia. Assumes zero effect from study site. p values are derived from a Cox model.

31 patients had recurrent parasitaemia by day 42, with a cumulative risk of 1·4% (95% CI 0·9–2·1) at day 28 and 4·9% (3·1–7·7) at day 42. When patients treated with chloroquine plus early primaquine were added to the previous Cox regression model, the addition of early primaquine was associated with a reduction in the rate of recurrent parasitaemia (AHR 0·10, 95% CI 0·05–0·17; p<0·0001; figure 4; appendix p 29). This reduction did not vary significantly with time; early primaquine was associated with a reduced rate of recurrence up to day 21 (AHR 0·07, 95% CI 0·03–0·18; p<0·0001) and between day 22 and day 42 (0·10, 0·06–0·18; p<0·0001). In a multivariable model of patients only treated with chloroquine plus early primaquine, neither primaquine dose nor chloroquine dose were significantly associated with a lower rate of recurrent parasitaemia (appendix p 30).

## Discussion

In this pooled analysis of individual patient data, a high proportion of patients, especially older males, received a suboptimal dose of chloroquine (<25 mg/kg); increasing the total mg/kg chloroquine dose reduced early recurrences if primaquine was not given, especially in children younger than 5 years; and, the risk of early recurrent parasitaemia was markedly reduced by co-administration of primaquine.

Increasing reports of declining chloroquine efficacy have highlighted the need for alternative treatment strategies for *P vivax*.^5^ In countries where there are high levels of chloroquine resistance, national guidelines have changed to ACT as first-line therapy for *P vivax*.^52^ Other countries have included primaquine as adjunctive therapy to prevent *P vivax* relapses, with the added benefit of providing additional blood schizontocidal activity.^12^, ^53^ However, the risk of substantial haemolysis, coupled with poor adherence, have prevented widespread effective implementation.^53^, ^54^ The results of this individual pooled data meta-analysis suggest that in the absence of primaquine, an increased dose of chloroquine would also decrease *P vivax* recurrence substantially in children younger than 5 years.

Previous pharmacokinetic studies have shown that chloroquine is under-dosed in children and have suggested that an increase in the chloroquine dose or dosing based on body surface area would be more appropriate and effective.^55^, ^56^, ^57^, ^58^ In children younger than 2 years, approximately twice the dose of chloroquine was required to reach the same chloroquine blood concentration as children aged 10–14 years.^55^ In addition, Añez and colleagues^58^ found that children had the greatest variation between dose per kg of bodyweight and theoretical dose calculated by body surface area. Chloroquine blood concentrations are also lowest in children, in whom the risk of recurrence is greatest.^58^

Our data are in keeping with these findings and suggest that increasing the total chloroquine dose from 25 mg/kg to 30 mg/kg in children younger than 5 years would decrease the risk of early recurrence by more than 40% if chloroquine was used alone. Although increasing the target dose might reduce tolerability, substantial data support the safety of 30 mg/kg in children. In Guinea-Bissau, chloroquine doses of 50 mg/kg against drug-resistant *Plasmodium falciparum* were well tolerated in children younger than 15 years.^59^, ^60^, ^61^, ^62^ Even higher doses have been used for amoebic liver abscess (21 mg/kg daily for 3 weeks)^63^ and *Giardia lamblia* (10 mg/kg twice daily for 5 days).^64^ Our pooled analysis did not include a comprehensive safety analysis, but, reassuringly, the risk of vomiting after chloroquine treatment was low and was not associated with chloroquine dose.

Current molecular analyses cannot differentiate reliably between the three causes of recurrent *P vivax* parasitaemia: recrudescence, relapse, and new infections.^65^ Hence, increasing the dose of chloroquine might simply provide a prolonged period of chemoprophylaxis, delaying recurrent infection rather than preventing recrudescence. Although this prolonged chemoprophylaxis is likely to account for some of the reduction in recurrences after a higher chloroquine dose, two factors suggest that there is also a reduction in the risk of recrudescence. First, regions with long relapse periodicity have a low risk of relapse within 6 weeks of treatment, increasing the likelihood that recurrences during this period are attributable to recrudescence. A subgroup analysis of patients from long relapse periodicity regions showed that a higher dose of chloroquine was protective against recurrence even in this setting (AHR per 5 mg/kg increase 0·63, 95% CI 0·42–0·96; p=0·031; appendix p 31). Second, the reduction in rate of recurrence associated with chloroquine dose in children younger than 5 years did not vary significantly over the follow-up period. By contrast, for older patients, the hazard ratio decreased after day 21 of follow-up compared with earlier. Between days 7 and 21, recurrences are more likely to be due to recrudescence, compared with relapses or new infections after this time.^2^, ^66^ Hence, in older patients, a higher chloroquine dose might afford greater prevention of relapse or new infection between days 22 and 42, but have minimal effect on true recrudescent infections. Conversely, in younger patients, a higher chloroquine dose probably also reduces recrudescent infections as a result of relative under-dosing of chloroquine despite delivery of the recommended chloroquine dose in this age group. Although our study design did not allow us to establish conclusively whether an increased dose of chloroquine prevents or delays parasite recurrence, either response is likely to be of substantial clinical benefit to the patient. Both responses allow greater time for haematological recovery after the initial infection, a reduced risk of cumulative anaemia, and thus the potential to reduce associated morbidity and mortality.^6^ However, prospective studies with prolonged follow-up are warranted.

The addition of primaquine to chloroquine reduced early recurrences before day 42 by 90% compared with chloroquine alone; probably in large part as a result of prevention of early relapse related to primaquine. However, addition of primaquine probably also reduces recrudescence through its blood schizontocidal activity, potentially in patients with low-grade chloroquine resistance. In the current pooled analysis, the reduction with chloroquine and primaquine did not vary before and after day 21, consistent with a reduction in both recrudescences and relapses.

Delayed parasite clearance predicts treatment failure in *P falciparum* malaria.^66^, ^67^, ^68^ Similar associations have been described in *P vivax*.^5^ In the current study, we confirm that delayed parasite clearance is associated with a higher risk of recurrence at day 28, consistent with an association with recrudescence. Although the specificity of persistent parasitaemia on day 3 for predicting risk of recurrence was 95·8%, the positive predictive value was only 23·0% (appendix p 32), showing the difficulty in using delayed parasite clearance as a measure of an individual's risk of recurrence. However, if parasite clearance was delayed until day 3, there was a three-times increased risk of recurrence at day 28. This association between delayed parasite clearance and recurrence is a potential parameter for identifying sites of possible chloroquine resistance, since this approach would avoid the confounding effect of relapses and reinfections that currently cannot be avoided in formal antimalarial efficacy studies.

Our study has several limitations. First, the analysis only included about 20% of patients from the clinical trials targeted. However, a sensitivity analysis in which one study site was removed at a time revealed no apparent bias relating to individual study sites that were included, and baseline characteristics of patients included had similar characteristics to those from all targeted studies (appendix p 21). Second, the number of tablets given was only available for about 60% of patients, with the remainder extrapolated from the protocol and assuming complete adherence. However, when the method used to calculate dose was included in the multivariable analyses, the results remained unchanged (data not shown).

In summary, although the risk of early recurrence of *P vivax* after chloroquine monotherapy is high, it can be reduced by a modest increase in the dose of chloroquine, particularly in children younger than 5 years, and by the additional administration of primaquine. As reports of chloroquine treatment failure for *P vivax* increase, we recommend that the dose of chloroquine be increased to 30 mg/kg in children younger than 5 years, and health-care providers should be encouraged to provide adjunctive primaquine radical therapy to reduce the risk of both recrudescent and relapsing infections. Alternatively, a universal policy of ACT for uncomplicated malaria, with additional primaquine for vivax malaria, should be considered in regions where there is a high risk of recurrent *P vivax* after chloroquine treatment.
