# Supplementary material for: The effect of chloroquine dose and primaquine on Plasmodium vivax recurrence: a WorldWide Antimalarial Resistance Network systematic review and individual patient pooled meta-analysis
Source: Lancet Infect Dis. 2018 Sep;18(9):1025–34. doi: 10.1016/S1473-3099(18)30348-7 (PMC6105624; doi:10.1016/S1473-3099(18)30348-7)
Supplement: Supplementary appendix [file mmc1.pdf]

THE LANCET Infectious Diseases

Supplementary webappendix

This webappendix formed part of the original submission and has been peer reviewed. We post it as supplied by the authors.

Supplement to: Commons RJ, Simpson JA, Thriemer K, et al. The effect of chloroquine dose and primaquine on Plasmodium vivax recurrence: a WorldWide Antimalarial Resistance Network systematic review and individual patient pooled meta-analysis. *Lancet Infect Dis* 2018; published online July 19. http://dx.doi.org/10.1016/ S1473-3099(18)30348-7.

APPENDIX

Commons, R.J., et al: **The effect of chloroquine dose and primaquine on *Plasmodium vivax* recurrence: a WorldWide Antimalarial Resistance Network systematic review and individual patient pooled meta-analysis**

	Page
**PRISMA-IPD Checklist**	2
**Box 1.** Search Strategy	6
**Study sites for clinical trials:**	
- **Figure S1.** Asia-Pacific Region	7
- **Figure S2.** Africa and Europe	8
- **Figure S3.** The Americas	9
**Figure S4**. Histogram of drug dosing	10
**Figure S5.** Mg/kg drug dosing of chloroquine by bodyweight in patients receiving chloroquine alone	11
**Figure S6.** Risk of recurrence by study at day 28 and 42 in patients receiving chloroquine alone	12
**Table S1.** Reasons for studies not being included in analysis	13
**Table S2.** Studies included in the analysis	14
**Table S3.** Study sites included in the analysis	16
**Table S4.** Studies targeted for the analysis but not included	18
**Table S5.** Comparison of baseline characteristics between included and targeted studies	21
**Total drug dosing by baseline characteristic:**	
- **Table S6A.** Patients receiving chloroquine alone	22
- **Table S6B.** Patients receiving chloroquine and early primaquine	23
**Table S7.** Risk factors for vomiting in patients treated with chloroquine alone	24
**Table S8.** Multivariable models for rate of *P. vivax* recurrence between day 7 and 42 in patients receiving chloroquine alone aged less than five years, five to less than 15 years or greater than or equal to 15 years	25
**Table S9.** Risk factors for patent parasitaemia on day 1 in patients receiving chloroquine alone	26
**Table S10.** Multivariable model evaluating the relationship between day of parasite clearance and rate of *P. vivax* recurrence between day 7 and 28 in patients receiving chloroquine alone	27
**Table S11.** Planned primaquine regimens in patients receiving chloroquine and early primaquine	28
**Table S12.** Multivariable model for rate of *P. vivax* recurrence between day 7 and 42 in patients receiving chloroquine alone or chloroquine and early primaquine	29
**Table S13.** Rate of *P. vivax* recurrence between day 7 and 42 in the subgroup of patients receiving chloroquine and early primaquine	30
**Table S14.** Multivariable model for rate of *P. vivax* recurrence between day 7 and 42 in patients aged less than five years receiving chloroquine alone in long relapse periodicity regions	31
**Table S15.** Accuracy of presence of parasite positivity in determining risk of recurrence prior to day 28 in patients receiving chloroquine alone	32
**Sensitivity analyses for the of rate of *P. vivax* recurrence between day 7 and 42:**	
- **Table S16.** Overall model of patients that received chloroquine alone	33
- **Table S17.** Patients receiving chloroquine alone aged less than 5 years	34
- **Table S18.** Patients receiving chloroquine alone aged five to less than 15 years	35
- **Table S19.** Patients receiving chloroquine alone aged 15 years or more	36
- **Table S20.** Patients receiving chloroquine alone or chloroquine and early primaquine	37
**References of all studies not included in analysis**	38

PRISMA-IPD Checklist of items to include when reporting a systematic review and meta-analysis of individual participant data (IPD)

PRISMA-IPD Section/topic	Item No	Checklist item	Reported on page
**Title**			
Title	1	Identify the report as a systematic review and meta-analysis of individual participant data.	1
**Abstract**			
Structured summary	2	Provide a structured summary including as applicable:	
		**Background**: state research question and main objectives, with information on participants, interventions, comparators and outcomes.	4
		**Methods**: report eligibility criteria; data sources including dates of last bibliographic search or elicitation, noting that IPD were sought; methods of assessing risk of bias.	4
		**Results**: provide number and type of studies and participants identified and number (%) obtained; summary effect estimates for main outcomes (benefits and harms) with confidence intervals and measures of statistical heterogeneity. Describe the direction and size of summary effects in terms meaningful to those who would put findings into practice.	4
		**Discussion:** state main strengths and limitations of the evidence, general interpretation of the results and any important implications.	4
		**Other:** report primary funding source, registration number and registry name for the systematic review and IPD meta-analysis.	4
**Introduction**			
Rationale	3	Describe the rationale for the review in the context of what is already known.	6
Objectives	4	Provide an explicit statement of the questions being addressed with reference, as applicable, to participants, interventions, comparisons, outcomes and study design (PICOS). Include any hypotheses that relate to particular types of participant-level subgroups.	6
**Methods**			
Protocol and registration	5	Indicate if a protocol exists and where it can be accessed. If available, provide registration information including registration number and registry name. Provide publication details, if applicable.	7 & 8
Eligibility criteria	6	Specify inclusion and exclusion criteria including those relating to participants, interventions, comparisons, outcomes, study design and characteristics (e.g. years when conducted, required minimum follow-up). Note whether these were applied at the study or individual level i.e. whether eligible participants were included (and ineligible participants excluded) from a study that included a wider population than specified by the review inclusion criteria. The rationale for criteria should be stated.	7
Identifying studies - information sources	7	Describe all methods of identifying published and unpublished studies including, as applicable: which bibliographic databases were searched with dates of coverage; details of any hand searching including of conference proceedings; use of study registers and agency or company databases; contact with the original research team and experts in the field; open adverts and surveys. Give the date of last search or elicitation.	7
Identifying studies - search	8	Present the full electronic search strategy for at least one database, including any limits used, such that it could be repeated.	Appendix

Study selection processes	9	State the process for determining which studies were eligible for inclusion.	7 (and ref 14)
Data collection processes	10	Describe how IPD were requested, collected and managed, including any processes for querying and confirming data with investigators. If IPD were not sought from any eligible study, the reason for this should be stated (for each such study).	7
		If applicable, describe how any studies for which IPD were not available were dealt with. This should include whether, how and what aggregate data were sought or extracted from study reports and publications (such as extracting data independently in duplicate) and any processes for obtaining and confirming these data with investigators.	7
Data items	11	Describe how the information and variables to be collected were chosen. List and define all study level and participant level data that were sought, including baseline and follow-up information. If applicable, describe methods of standardising or translating variables within the IPD datasets to ensure common scales or measurements across studies.	7 & 8
IPD integrity	A1	Describe what aspects of IPD were subject to data checking (such as sequence generation, data consistency and completeness, baseline imbalance) and how this was done.	7 (ref 15)
Risk of bias assessment in individual studies.	12	Describe methods used to assess risk of bias in the individual studies and whether this was applied separately for each outcome. If applicable, describe how findings of IPD checking were used to inform the assessment. Report if and how risk of bias assessment was used in any data synthesis.	8 & Appendix
Specification of outcomes and effect measures	13	State all treatment comparisons of interests. State all outcomes addressed and define them in detail. State whether they were pre-specified for the review and, if applicable, whether they were primary/main or secondary/additional outcomes. Give the principal measures of effect (such as risk ratio, hazard ratio, difference in means) used for each outcome.	7 & 8
Synthesis methods	14	Describe the meta-analysis methods used to synthesise IPD. Specify any statistical methods and models used. Issues should include (but are not restricted to):<br>• Use of a one-stage or two-stage approach.<br>• How effect estimates were generated separately within each study and combined across studies (where applicable).<br>• Specification of one-stage models (where applicable) including how clustering of patients within studies was accounted for.<br>• Use of fixed or random effects models and any other model assumptions, such as proportional hazards.<br>• How (summary) survival curves were generated (where applicable).<br>• Methods for quantifying statistical heterogeneity (such as I^2 and τ^2).<br>• How studies providing IPD and not providing IPD were analysed together (where applicable).<br>• How missing data within the IPD were dealt with (where applicable).	8
Exploration of variation in effects	A2	If applicable, describe any methods used to explore variation in effects by study or participant level characteristics (such as estimation of interactions between effect and covariates). State all participant-level characteristics that were analysed as potential effect modifiers, and whether these were pre-specified.	9
Risk of bias across studies	15	Specify any assessment of risk of bias relating to the accumulated body of evidence, including any pertaining to not obtaining IPD for particular studies, outcomes or other variables.	9 & Appendix

Additional analyses	16	Describe methods of any additional analyses, including sensitivity analyses. State which of these were pre-specified.	9
**Results**			
Study selection and IPD obtained	17	Give numbers of studies screened, assessed for eligibility, and included in the systematic review with reasons for exclusions at each stage. Indicate the number of studies and participants for which IPD were sought and for which IPD were obtained. For those studies where IPD were not available, give the numbers of studies and participants for which aggregate data were available. Report reasons for non-availability of IPD. Include a flow diagram.	10 & Appendix
Study characteristics	18	For each study, present information on key study and participant characteristics (such as description of interventions, numbers of participants, demographic data, unavailability of outcomes, funding source, and if applicable duration of follow-up). Provide (main) citations for each study. Where applicable, also report similar study characteristics for any studies not providing IPD.	Appendix
IPD integrity	A3	Report any important issues identified in checking IPD or state that there were none.	10 & Fig 1
Risk of bias within studies	19	Present data on risk of bias assessments. If applicable, describe whether data checking led to the up-weighting or down-weighting of these assessments. Consider how any potential bias impacts on the robustness of meta-analysis conclusions.	10 & Appendix
Results of individual studies	20	For each comparison and for each main outcome (benefit or harm), for each individual study report the number of eligible participants for which data were obtained and show simple summary data for each intervention group (including, where applicable, the number of events), effect estimates and confidence intervals. These may be tabulated or included on a forest plot.	10; Appendix
Results of syntheses	21	Present summary effects for each meta-analysis undertaken, including confidence intervals and measures of statistical heterogeneity. State whether the analysis was pre-specified, and report the numbers of studies and participants and, where applicable, the number of events on which it is based.	10-12
		When exploring variation in effects due to patient or study characteristics, present summary interaction estimates for each characteristic examined, including confidence intervals and measures of statistical heterogeneity. State whether the analysis was pre-specified. State whether any interaction is consistent across trials.	10-12
		Provide a description of the direction and size of effect in terms meaningful to those who would put findings into practice.	10-12
Risk of bias across studies	22	Present results of any assessment of risk of bias relating to the accumulated body of evidence, including any pertaining to the availability and representativeness of available studies, outcomes or other variables.	10 & Appendix
Additional analyses	23	Give results of any additional analyses (e.g. sensitivity analyses). If applicable, this should also include any analyses that incorporate aggregate data for studies that do not have IPD. If applicable, summarise the main meta-analysis results following the inclusion or exclusion of studies for which IPD were not available.	Appendix
**Discussion**			
Summary of evidence	24	Summarise the main findings, including the strength of evidence for each main outcome.	13-15
Strengths and limitations	25	Discuss any important strengths and limitations of the evidence including the benefits of access to IPD and any limitations arising from IPD that were not available.	15

Conclusions	26	Provide a general interpretation of the findings in the context of other evidence.	13-15
Implications	A4	Consider relevance to key groups (such as policy makers, service providers and service users). Consider implications for future research.	13-15
**Funding**			
Funding	27	Describe sources of funding and other support (such as supply of IPD), and the role in the systematic review of those providing such support.	9

© Reproduced with permission of the PRISMA IPD Group, which encourages sharing and reuse for non-commercial purposes

Box 1. Search strategy

Search strategy

All prospective *P. vivax* antimalarial clinical trials with a minimum of 28 days follow up, published between Jan 1, 2000 and March 22, 2017 were identified by the application of the key terms (listed below) through Medline (Pubmed), Web of Science, Embase and the Cochrane Database of Systematic Reviews. Abstracts of all references containing any mention of antimalarial drugs were manually checked to confirm prospective clinical trials, with review of full text when needed. Studies on prevention, prophylaxis, reviews, animal studies, patients with severe malaria, where schizontocidal treatment was unsupervised or where data were extracted retrospectively from medical records outside of a planned trial were excluded. The year of the study was taken as the year in which the paper was published, although the start and end date of patient enrolment were also recorded. The review process was undertaken by two independent investigators who also performed data extraction (RJC and RNP), and is documented in more detail in Commons *et al*, Int J Parasitol Drug Drug Res 2017.[14]

Key terms:

Literature search (conducted March 2017) with the following key terms (version undertaken in Pubmed): (malaria OR plasmod*) AND (amodiaquine OR atovaquone OR artemisinin OR arteether OR artesunate OR artemether OR artemotil OR azithromycin OR artekin OR chloroquine OR chlorproguanil OR cycloguanil OR clindamycin OR coartem OR dapsone OR dihydroartemisinin OR duo-cotecxin OR doxycycline OR halofantrine OR lumefantrine OR lariam OR malarone OR mefloquine OR naphthoquine OR naphthoquinone OR piperaquine OR primaquine OR proguanil OR pyrimethamine OR pyronaridine OR quinidine OR quinine OR riamet OR sulphadoxine OR tetracycline OR tafenoquine).

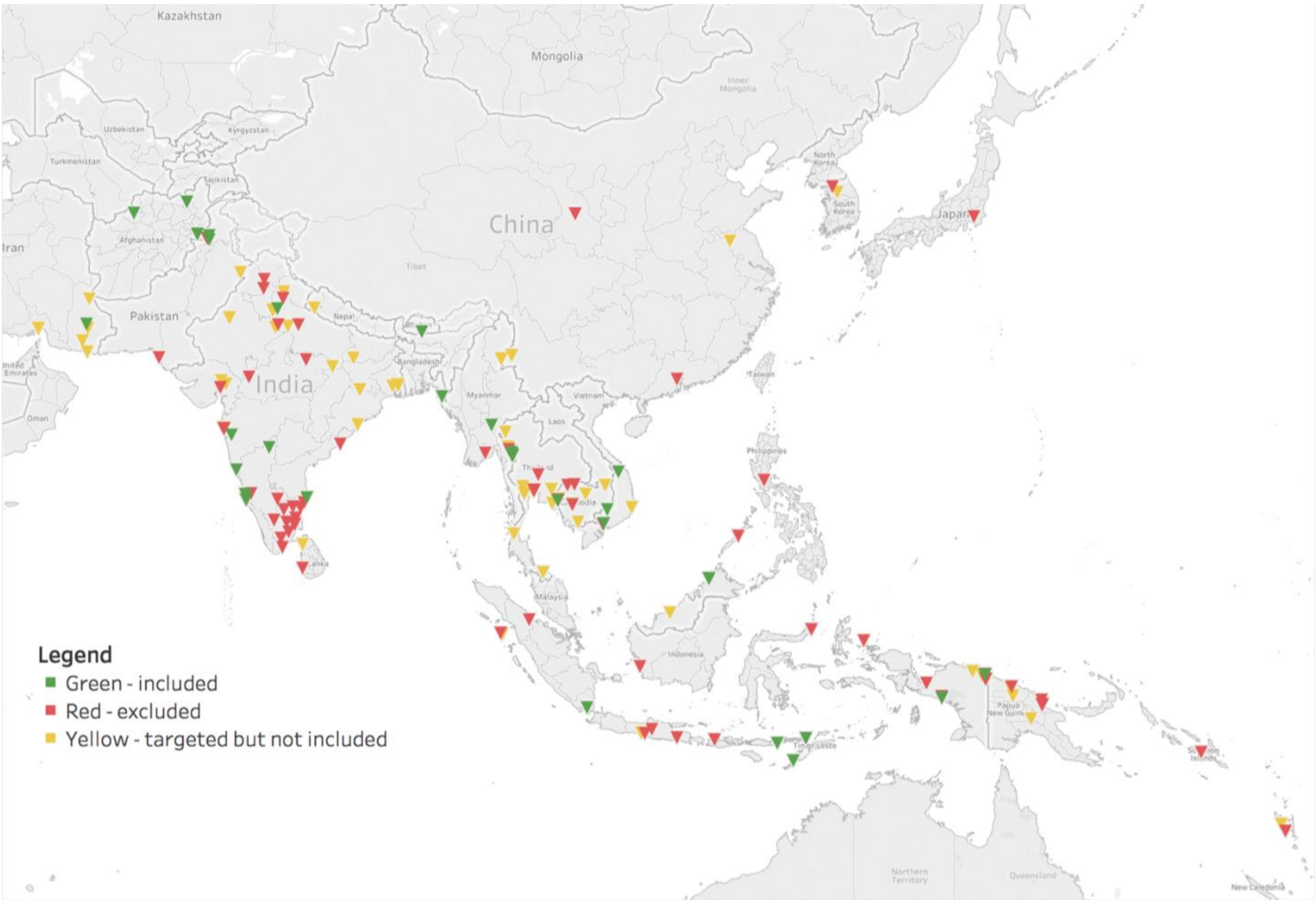

Figure S1. Study sites for clinical trials – Asia-Pacific Region

Studies of prisoners, travelers and soldiers are marked at the location of the study site.

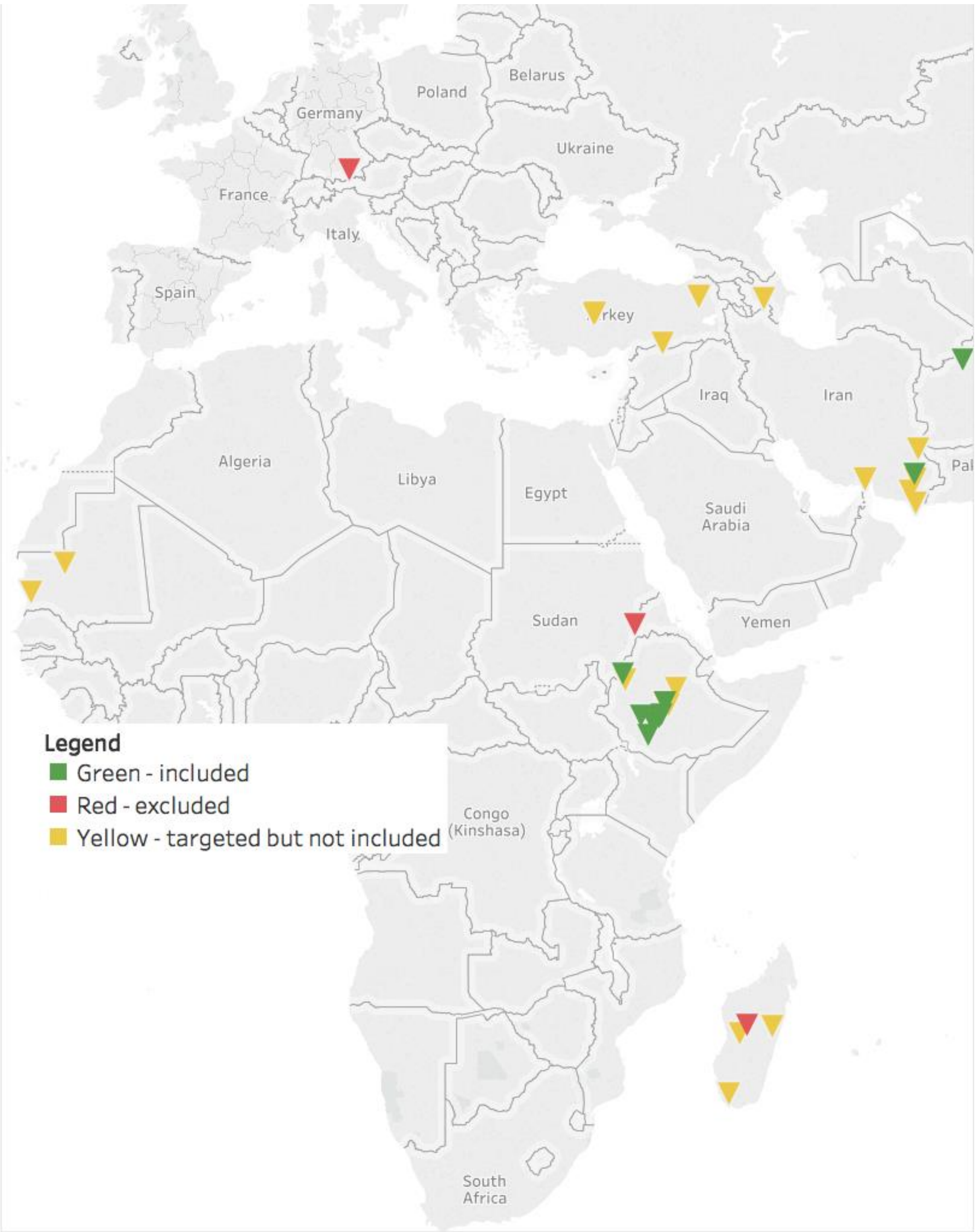

Figure S2. Study sites for clinical trials – Africa and Europe

Studies of prisoners, travelers and soldiers are marked at the location of the study site.

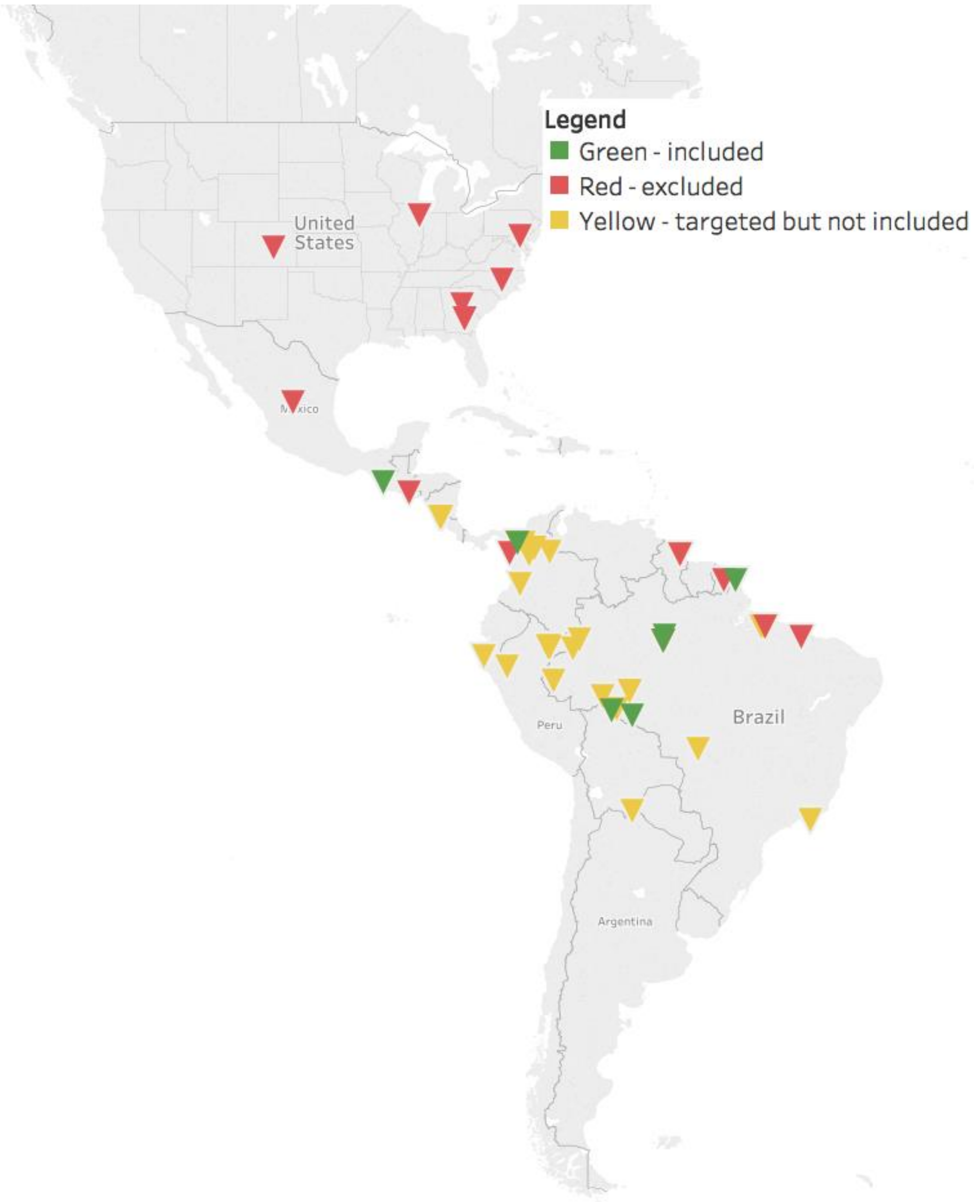

Figure S3. Study sites for clinical trials – The Americas

Studies of prisoners, travelers and soldiers are marked at the location of the study site.

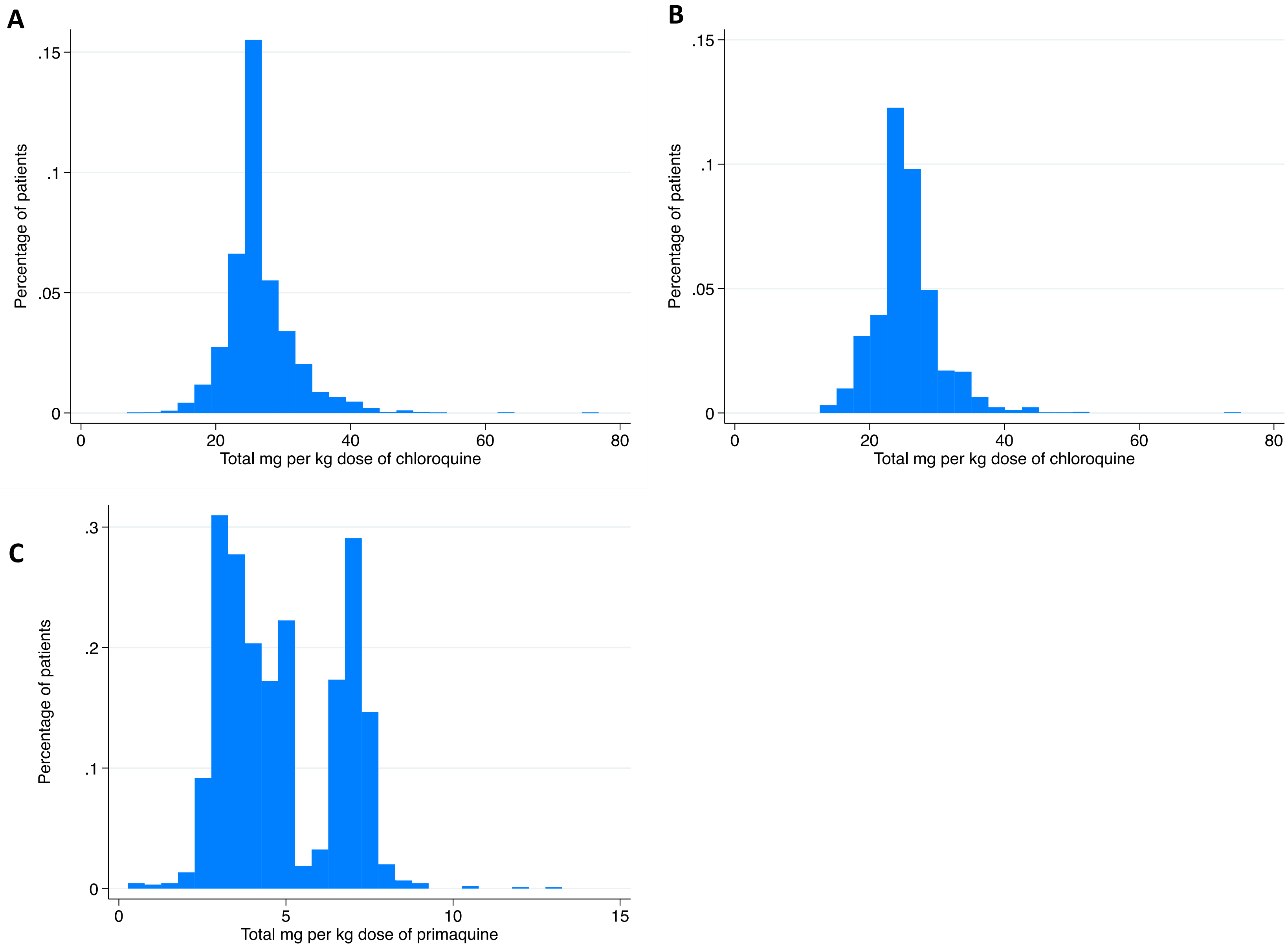

Figure S4. Histogram of drug dosing for (A) chloroquine in patients receiving chloroquine alone, (B) chloroquine in patients receiving chloroquine plus early primaquine and (C) primaquine in patients receiving chloroquine plus early primaquine.

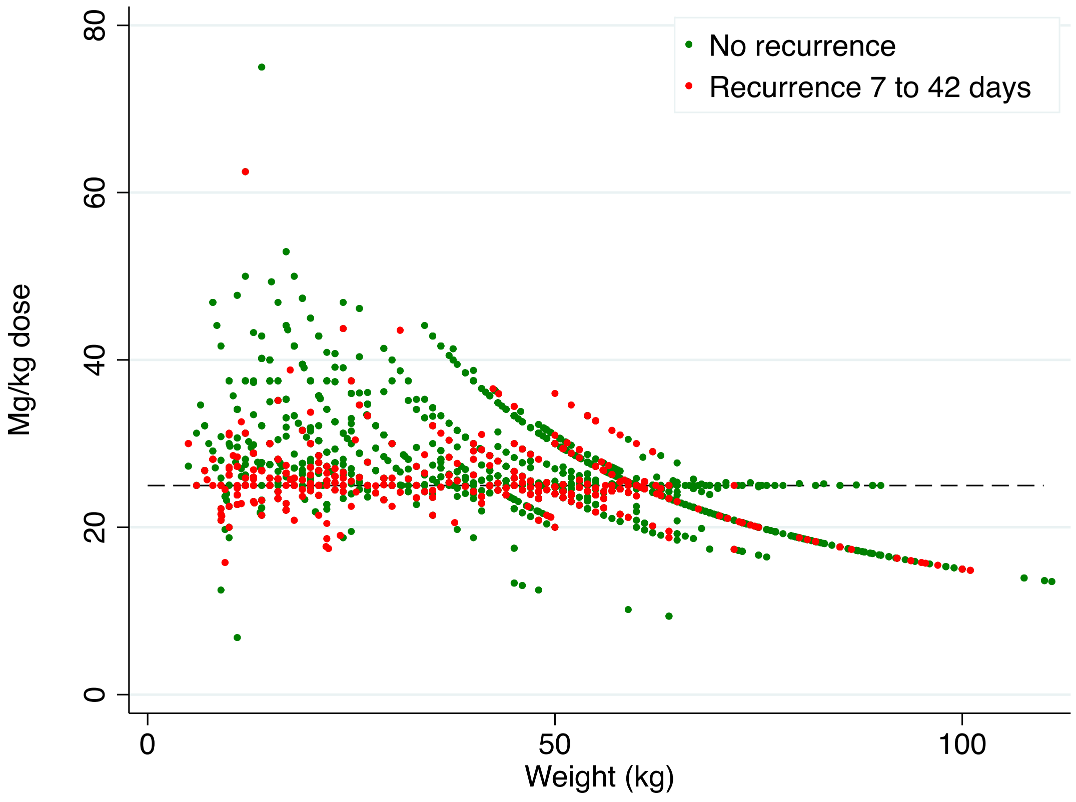

Figure S5. Mg/kg drug dosing of chloroquine by bodyweight in patients receiving chloroquine alone.
Dashed line: 25 mg/kg dose of chloroquine (current recommended dose).

A. Day 28

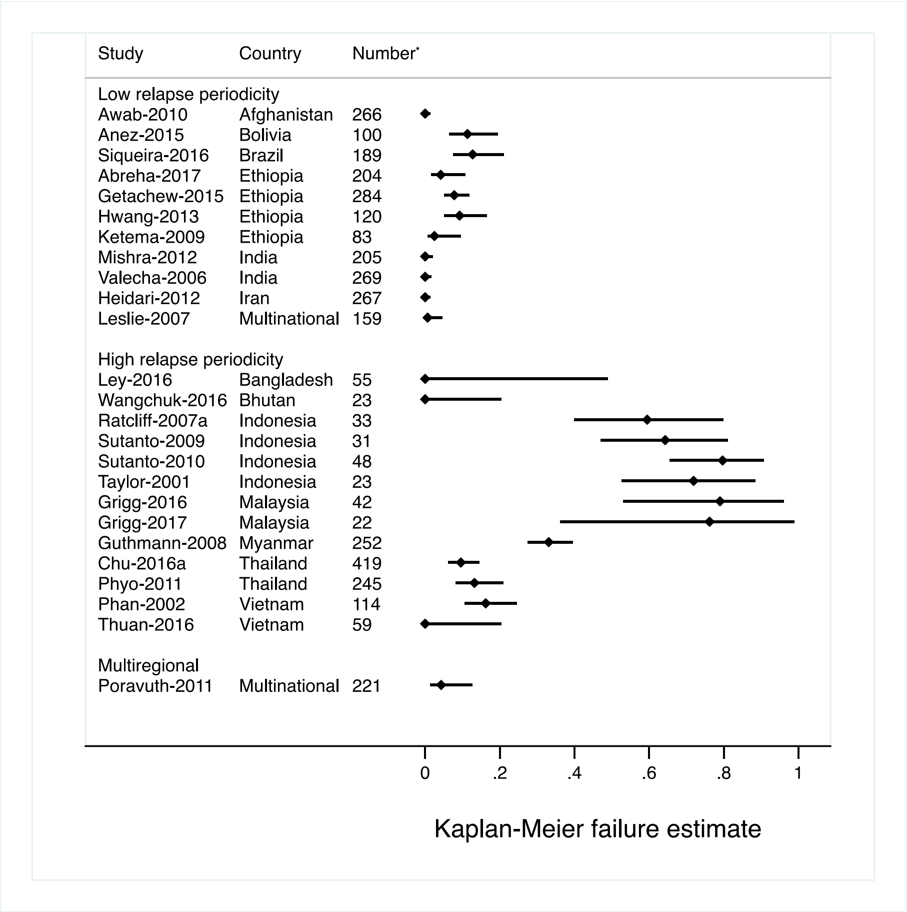

B. Day 42

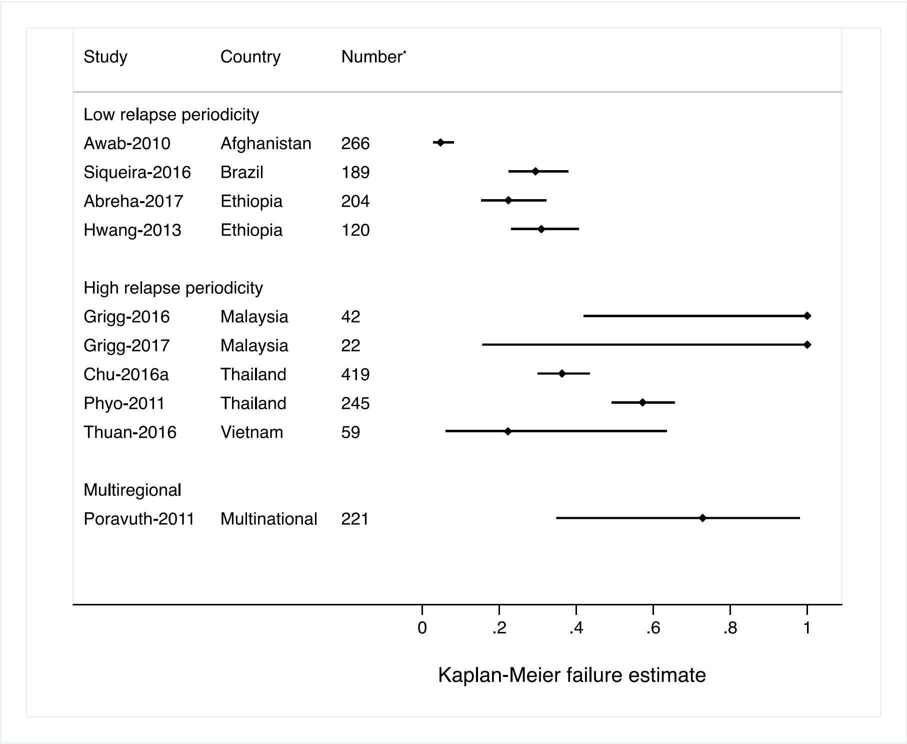

Figure S6. Risk of recurrence by study at day 28 and 42 in patients receiving chloroquine alone

* Number refers to the total number of patients available for analysis from the study; Where a Kaplan-Meier failure estimate for day 28 was not available due to no failures having occurred, confidence intervals were generated using Wilson's procedure for patients followed to day 28. Barber *et al* did not have any failures, or any patients followed until day 28.[33]

Table S1. Reasons for studies not being included in analysis

Reason	Number of studies	Studies*
Published pre-2000	64	13, 69-131
No chloroquine treatment arm	28	132-159
Adjunctive drug(s) used	4	160-163
Study of pregnant women	2	164,165
Data not available	8	166-173
Investigators unable to be contacted	15	174-188
Missing minimum data for inclusion	7	189-195
Initial investigator response but no data provided	7	196-202
No response from investigators	64	203-266

* References of studies not included are provided in Appendix, page 38

Table S2. Studies included in analysis

Author-year	Country	Recruitment	Included in study			Follow up (days)	Treatment arms	Supervision	Patients enrolled	Patients available	Patients included	CQ dosing schedule	CQ target (mg/kg)	CQ dose calculation method	Primaquine dosing	Timing of primaquine	Published
			<5yrs	5-15yrs	>15yrs												
Taylor-2001[19]	Indonesia	1995-1998	No	No	Yes	28	CQ, CQ+Doxy, Doxy	Full	63	64	23	Day 1: 10mg/kg, Day 2: 10mg/kg, Day 3: 5mg/kg	25	Per protocol	14day-High Dose	End of Study	Yes
Phan-2002[20]	Vietnam	1997-2000	No	No	Yes	28	CQ, Art	Full	226	232	114	Day 1: 15mg/kg, Day 2: 5mg/kg, Day 3: 5mg/kg	25	Per protocol	5day-Very Low Dose	End of Study	Yes
Valecha-2006[21]	India	2002	Yes	Yes	Yes	28	CQ	Full	287	287	269	25mg/kg total 3 days	25	Tablet count	-	-	Yes
Leslie-2007[22]	Afghanistan, Pakistan	2004-2006	Yes	Yes	Yes	28	CQ, Chlorproguanil-dapsone, SP	Full	767	767	159	25mg/kg over 3 days	25	Per protocol	-	-	Yes
Ratcliff-2007a[23]	Indonesia	2004	Yes	Yes	Yes	28	CQ	Full	40	61	33	25mg/kg over 3 days	25	Tablet count	14day-Low Dose	End of Study	Yes
Guthmann-2008[24]	Myanmar	2002-2003	Yes	Yes	Yes	28	CQ	Full	252	252	252	Day 1: 10mg/kg, Day 2: 10mg/kg, Day 3: 5mg/kg	25	Per protocol	-	-	Yes
Leslie-2008[25]	Pakistan	2004-2007	Yes	Yes	Yes	365	CQ, CQ+PQ, CQ+PQ	Full	200	210	122	Day 1: 10mg/kg, Day 2: 10mg/kg, Day 3: 5mg/kg	25	Per protocol	None, 14 day High Dose; weekly over 8 weeks (excluded)	Day 0	Yes
Ketema-2009[26]	Ethiopia	2007-2008	No	Yes	Yes	28	CQ	Full	84	84	83	Day 1: 10mg/kg, Day 2: 10mg/kg, Day 3: 5mg/kg	25	Tablet count	-	-	Yes
Sutanto-2009[27]	Indonesia	2001-2002	Yes	Yes	Yes	28	CQ	Full	36	31	31	Day 1: 10mg/kg, Day 2: 10mg/kg, Day 3: 5mg/kg	25	Tablet count	Primaquine	End of Study	Yes
Awab-2010[28]	Afghanistan	2007-2009	Yes	Yes	Yes	56	CQ, DP	Full	536	536	266	Day 1: 10mg/kg, Day 2: 10mg/kg, Day 3: 5mg/kg	25	Tablet count	-	-	Yes
Sutanto-2010[29]	Indonesia	2002	Yes	Yes	Yes	28	CQ	Full	31	49	48	Day 1: 10mg/kg, Day 2: 10mg/kg, Day 3: 5mg/kg	25	Tablet count	-	-	Yes
Phyo-2011[30]	Thailand	2007-2008	Yes	Yes	Yes	63	CQ, DP	Full	492	498	245	Day 1: 10mg/kg, Day 2: 10mg/kg, Day 3: 5mg/kg	25	Tablet count	14day-High Dose	End of Study	Yes
Poravuth-2011[31]	Multicentred	2007-2008	Yes	Yes	Yes	42	CQ, AS+Pyr	Full	456	456	221	Day 1: 10mg/kg, Day 2: 10mg/kg, Day 3: 5mg/kg	25	Tablet count	14day-Low Dose	Day 28	Yes
Heidari-2012[51]	Iran	2010	Yes	Yes	Yes	28	CQ	Full	285	270	267	Day 1: 10mg/kg, Day 2: 10mg/kg, Day 3: 5mg/kg	25	Per protocol	Weekly over 8 weeks	End of Study	Yes
Mishra-2012[32]	India	2009-2010	Yes	Yes	Yes	28	CQ	Full	210	211	205	25mg/kg over 3 days	25	Tablet count	14day-Low Dose	End of Study	Yes
Barber-2013[33]	Malaysia	2010-2011	No	Yes	Yes	43	CQ+PQ, varied	Not stated	43	86	13	Hour 0: 10mg/kg, Hour 6: 5mg/kg, Hour 24: 5mg/kg, Hour 48: 5mg/kg	25	Tablet count	14day-High Dose	Day 0	Yes
Hwang-2013[34]	Ethiopia	2009-2010	Yes	Yes	Yes	42	CQ, AL	Full CQ; Partial AL	242	242	120	Day 1: 10mg/kg, Day 2: 10mg/kg, Day 3: 5mg/kg	25	Per protocol	-	-	Yes
Marques-2014[35]	Brazil	2007-2008	No	Yes	Yes	28	CQ+PQ	Full	135	154	135	Day 1: 10mg/kg, Day 2: 7.5mg/kg, Day 3: 7.5mg/kg	25	Per protocol	7 day-Low Dose	Day 0	Yes
Anez-2015[36]	Bolivia	2011	No	Yes	Yes	28	CQ	Full	100	100	100	Day 1: 10mg/kg, Day 2: 10mg/kg, Day 3: 5mg/kg	25	Tablet count	7 day-Low Dose	Day 28	Yes
Getachew-2015[37]	Ethiopia	2010-2013	Yes	Yes	Yes	28	CQ	Full	288	288	284	Day 1: 10mg/kg, Day 2: 10mg/kg, Day 3: 5mg/kg	25	Tablet count	-	-	Yes
Gomes-2015[38]	Brazil	2011-2012	No	No	Yes	28	CQ+PQ	Partial CQ and PQ	103	94	93	Day 1: 10mg/kg, Day 2: 7.5mg/kg, Day 3: 7.5mg/kg	25	Per protocol	7 day-Low Dose	Day 0	Yes
Gonzalez-Ceron -2015[39]	Mexico	2008-2009	Yes	Yes	Yes	365	(1) CQ+PQ, (2) CQ+PQ	Full	153	159	88 (arm 2 excluded)	(1) Day 1: 10mg/kg, Day 2: 10mg/kg, Day 3: 5mg/kg; (2) 80mg/kg intermittently over 8 months	(1) 25; (2) 80	Tablet count	14 day-Low Dose; intermittently over 8 months (excluded)	Day 0	Yes
Lidia-2015[40]	Indonesia	2013	No	No	Yes	42	CQ+PQ, DP+PQ	Full	51	51	26	Day 1: 10mg/kg, Day 2: 10mg/kg, Day 3: 5mg/kg	25	Tablet count	14 day-Low Dose	Day 0	Yes

Rishikesh-2015[41]	India	2012-2014	No	No	Yes	28	CQ+PQ	Full CQ; Partial PQ	125	125	124	25mg/kg total 3 days	25	Tablet count	14day-Low Dose; weekly over 8 weeks (excluded)	Day 0	Yes
Thanh-2015[42]	Vietnam	2009-2011	Yes	Yes	Yes	28	CQ+PQ	Full	260	260	260	Day 1: 10mg/kg, Day 2: 10mg/kg, Day 3: 5mg/kg	25	Tablet count	10day-High Dose	Day 0	Yes
Grigg-2016[43]	Malaysia	2012-2014	Yes	Yes	Yes	42	AS+MQ+PQ, CQ+PQ	Full	103	103	42	Hour 0: 10mg/kg, Hour 6: 5mg/kg, Hour 24: 5mg/kg, Hour 48: 5mg/kg	25	Tablet count	14day-High Dose	Day 28	Yes
Ley-2016[44]	Bangladesh	2014-2015	Yes	Yes	Yes	30	AL+PQ, CQ+PQ	Partial AL; Full CQ; Partial PQ	55	66	55	Day 1: 10mg/kg, Day 2: 10mg/kg, Day 3: 5mg/kg	25	Tablet count	14day-Low Dose	Day 2	Yes
Pereira-2016[45]	Brazil	2013-2015	No	No	Yes	28	CQ+PQ	Partial CQ and PQ	88	88	86	Day 1: 10mg/kg, Day 2: 7.5mg/kg, Day 3: 7.5mg/kg	25	Tablet count	7-9 day Low-Dose	Day 0	Yes
Saravu-2016[46]	India	2012-2015	No	No	Yes	28	CQ+PQ, CQ+PQ(weekly)	Partial CQ and PQ	161	161	136	25mg/kg total 3 days	25	Tablet count	14 day-Low Dose; weekly over 8 weeks (excluded)	Day 0	Yes
Thuan-2016[47]	Vietnam	2013-2014	Yes	Yes	Yes	63	CQ, DP	Full	128	128	59	25mg/kg total given at hour 0, 8, 24, 32, 48	25	Tablet count	14day-Low Dose	End of Study	Yes
Wangchuk-2016[48]	Bhutan	2013-2015	Yes	Yes	Yes	365	CQ+PQ	Full	24	28	23	Day 1: 10mg/kg, Day 2: 10mg/kg, Day 3: 5mg/kg	25	Tablet count	14day-Low Dose	Day 28	Yes
Zuluaga-Idarraga-2016[49]	Colombia	2012-2014	Yes	Yes	Yes	180	CQ+PQ	Full	87	87	85	Day 1: 10mg/kg, Day 2: 7.5mg/kg, Day 3: 7.5mg/kg	25	Tablet count	14 day-Low Dose	Day 0	Yes
Abreha-2017[12]	Ethiopia	2012-2016	Yes	Yes	Yes	365	AL, AL+PQ, CQ, CQ+PQ	Partial AL; Full CQ; Partial PQ	398	398	204	Day 1: 10mg/kg, Day 2: 10mg/kg, Day 3: 5mg/kg	25	Tablet count	14day-Low Dose	Day 2	After literature search
Chu-2017a	Thailand	2010-2012	Yes	Yes	Yes	365	CQ, CQ+PQ, AS	Full	645	645	419	Day 1: 10mg/kg, Day 2: 10mg/kg, Day 3: 5mg/kg	25	Per protocol	None, 14 day High Dose	Day 0	No
Chu-2017b	Thailand	2012-2014	Yes	Yes	Yes	365	DP+PQ, DP+PQ, CQ+PQ, CQ+PQ	Full	680	680	339	Day 1: 10mg/kg, Day 2: 10mg/kg, Day 3: 5mg/kg	25	Per protocol	7 day High-dose, 14 day High-dose	Day 0	No
Grigg-2017	Malaysia	2013-2016	No	Yes	Yes	42	CQ+PQ, variable	Not stated	57	57	22	Hour 0: 10mg/kg, Hour 6: 5mg/kg, Hour 24: 5mg/kg, Hour 48:5 mg/kg	25	Tablet count	14day-High Dose	Day 0	No
Siqueira-2017[50]	Brazil	2012-2013	Yes	Yes	Yes	42	AS+AQ, CQ	Full	380	380	189	Day 1: 10mg/kg, Day 2: 7.5mg/kg, Day 3: 7.5mg/kg	25	Tablet count	-	-	Yes

CQ – chloroquine; Doxy – doxycycline; Art – artemisinin; SP – Sulfadoxine-pyrimethamine; PQ – primaquine; AS – artesunate; Pyr – pyronaridine; AL – artemether-lumefantrine; DP – dihydroartemisinin-piperaquine

Table S3. Study sites included in analysis

Author-Year	Country	Study site	Latitude	Longitude	Year Start	Year End	MAP PvPR % (2010)*	Publication estimate of prevalence	Final prevalence classification	Region of relapse periodicity[17]	Final category of relapse periodicity†
Taylor-2001[19]	Indonesia	Papua	-2.56	140.72	1995	1998	0.0	Not stated	Low	12	High
Phan-2002[20]	Vietnam	Binh Thuan	10.82	106.73	1997	2000	0.5	Not stated	Low	10	High
Valecha-2006[21]	India	Chennai	13.06	80.25	2002	2002	2.0	Perennial; API 2001 - 5.2; 2002 - 5.9; 2003 - 6.2	Moderate	8	Low
Valecha-2006[21]	India	Gautam	28.76	77.63	2002	2002	2.6	Seasonal API 2001 - 1.88; 2002 - 0.23; 2003 - 1.08	Moderate	8	Low
Valecha-2006[21]	India	Navi Mumbai	18.44	73.51	2002	2002	4.3	Perennial; API 2002 - 3.69; 2003 - 2.27	High	8	Low
Leslie-2007[22]	Afghanistan	Jalalabad	34.43	70.46	2004	2006	1.4	Not stated	Low	11	Low
Leslie-2007[22]	Pakistan	Adizai	33.78	71.57	2004	2006	1.8	Not stated	Moderate	8	Low
Ratcliff-2007a[23]	Indonesia	Timika	-4.61	136.85	2004	2004	4.7	Unstable; 279 per 1000 per year	High	12	High
Guthmann-2008[24]	Myanmar	Dawei	19.26	96.68	2002	2003	4.6	Not stated	High	10	High
Leslie-2008[25]	Pakistan	Adizai	33.78	71.57	2004	2007	1.8	Seasonal	Moderate	8	Low
Leslie-2008[25]	Pakistan	Baghicha	35.59	75.33	2004	2007	0.6	Seasonal	Low	11	Low
Leslie-2008[25]	Pakistan	Khagan	34.78	73.53	2004	2007	1.2	Seasonal	Low	11	Low
Ketema-2009[26]	Ethiopia	Serbo	9.37	34.58	2007	2008	0.7	Seasonal; 3925 to 22938 cases in population of 329629 from 2002 to 2007	Low	7	Low
Sutanto-2009[27]	Indonesia	Alor	-8.25	124.75	2001	2002	2.9	Not stated	Moderate	10	High
Awab-2010[28]	Afghanistan	Jalalabad	34.43	70.46	2007	2009	1.4	Seasonal	Low	11	Low
Awab-2010[28]	Afghanistan	Maimana	35.92	64.82	2007	2009	0.6	Seasonal	Low	11	Low
Awab-2010[28]	Afghanistan	Taloqan	36.74	69.54	2007	2009	0.4	Seasonal	Low	11	Low
Sutanto-2010[29]	Indonesia	Lampung	-5.53	105.24	2002	2002	0.5	Not stated	Low	10	High
Phyo-2011[30]	Thailand	Mae Sot	16.72	98.58	2007	2008	4.0	Low EIR <1; seasonal transmission	High	10	High
Poravuth-2011[31]	Cambodia	Pailin	12.85	102.61	2007	2008	1.8	Not stated	Moderate	10	High
Poravuth-2011[31]	India	Mangalore	12.87	74.84	2007	2008	2.7	Not stated	Moderate	8	Low
Poravuth-2011[31]	Indonesia	Maumere	-8.62	122.21	2007	2008	1.9	Not stated	Moderate	10	High
Poravuth-2011[31]	Thailand	Mae Ramat	16.98	98.52	2007	2008	3.8	Not stated	Moderate	10	High
Poravuth-2011[31]	Thailand	Mae Sot	16.72	98.58	2007	2008	4.0	Not stated	High	10	High
Heidari-2012[51]	Iran	Sistan va Baluchistan	27.53	60.59	2010	2010	0.5	Seasonal	Low	8	Low
Mishra-2012[32]	India	Gulburga	17.39	76.87	2009	2010	2.9	Not stated	Moderate	8	Low
Mishra-2012[32]	India	Mangalore	12.91	74.86	2009	2010	3.2	Not stated	Moderate	8	Low
Mishra-2012[32]	India	North Goa	15.52	73.98	2009	2010	2.7	Not stated	Moderate	8	Low
Barber-2013[33]	Malaysia	Sabah	5.98	116.08	2010	2011	3.9	Not stated	Moderate	10	High
Hwang-2013[34]	Ethiopia	Bishoftu	8.74	38.99	2009	2010	1.4	Seasonal; parasite prevalence rates of 0.3 and 0.9% in all ages and 0.6% in Oromia in 5-18 year olds	Low	7	Low
Hwang-2013[34]	Ethiopia	Bulbula	7.72	38.65	2009	2010	2.2	Seasonal; parasite prevalence rates of 0.3 and 0.9% in all ages and 0.6% in Oromia in 5-18 year olds	Moderate	7	Low
Marques-2014[35]	Brazil	Manaus	-3.12	-60.02	2007	2008	6.9	Not stated	High	3	Low
Anez-2015[36]	Bolivia	Riberalta	-11.01	-66.06	2011	2011	4.9	Not stated	High	3	Low
Getachew-2015[37]	Ethiopia	Arba Minch	6.01	37.54	2010	2013	1.1	2012 API: 20.3	Low	7	Low
Getachew-2015[37]	Ethiopia	Halaba	7.49	38.19	2010	2013	1.9	2012 API: 81.8	Moderate	7	Low
Getachew-2015[37]	Ethiopia	Shone	7.14	37.95	2010	2013	1.8	2012 API: 41.0	Moderate	7	Low
Getachew-2015[37]	Ethiopia	Ziway	7.92	38.72	2010	2013	1.9	2012 API: 35.4	Moderate	7	Low

Gomes-2015[38]	Brazil	Oiapoque	3.84	-51.83	2011	2012	4.9	API 246 in 2011	High	3	Low
Gonzalez-Ceron -2015[39]	Mexico	Cacahoatan	15.09	-92.21	2008	2009	1.9	Hypoendemic; variable; API 0.579-1.523 for 2003 to 2007	Moderate	2	Low
Gonzalez-Ceron -2015[39]	Mexico	Fra Hidalgo	14.74	-92.23	2008	2009	1.4	Hypoendemic; variable; API 0.579-1.523 for 2003 to 2007	Low	2	Low
Gonzalez-Ceron -2015[39]	Mexico	Huehuetan	15.06	-92.33	2008	2009	1.7	Hypoendemic; variable; API 0.579-1.523 for 2003 to 2007	Moderate	2	Low
Gonzalez-Ceron -2015[39]	Mexico	Tapachula	14.91	-92.26	2008	2009	2.0	Hypoendemic; variable; API 0.579-1.523 for 2003 to 2007	Moderate	2	Low
Gonzalez-Ceron -2015[39]	Mexico	Tuxtla Chico	14.91	-92.15	2008	2009	2.1	Hypoendemic; variable; API 0.579-1.523 for 2003 to 2007	Moderate	2	Low
Lidia-2015[40]	Indonesia	Kupang	-10.18	123.61	2013	2013	0.9	Not stated	Low	10	High
Rishikesh-2015[41]	India	Manipal	13.36	74.79	2012	2014	3.2	Not stated	Moderate	8	Low
Thanh-2015[42]	Vietnam	Tra Leng	15.28	107.99	2009	2011	2.2	Perennial; prevalence variable within region from <5 to 30%	Moderate	10	High
Grigg-2016[43]	Malaysia	Kota Marudu	6.49	116.77	2012	2014	4.7	Not stated	High	10	High
Grigg-2016[43]	Malaysia	Kudat	6.89	116.85	2012	2014	3.8	Not stated	Moderate	10	High
Grigg-2016[43]	Malaysia	Pitas	6.71	117.03	2012	2014	4.7	Not stated	High	10	High
Ley-2016[44]	Bangladesh	Alikadam Upazilla	23.70	90.44	2014	2015	0.6	Not stated	Low	10	High
Pereira-2016[45]	Brazil	Rondonia	-11.51	-63.58	2013	2015	4.8	Not stated	High	3	Low
Saravu-2016[46]	India	Udupi taluk	13.48	74.71	2012	2015	2.6	API estimated at 2.5	Moderate	8	Low
Thuan-2016[47]	Vietnam	Bu Gia Map	12.04	107.05	2013	2014	2.4	High	Moderate	10	High
Thuan-2016[47]	Vietnam	Dak O	12.00	107.50	2013	2014	1.3	High	Moderate	10	High
Wangchuk-2016[48]	Bhutan	Pemagatshel	27.04	91.45	2013	2015	0.9	Low	Low	10	High
Wangchuk-2016[48]	Bhutan	Samdrupjongkhar	26.93	91.64	2013	2015	1.0	Low	Low	10	High
Wangchuk-2016[48]	Bhutan	Sarpang	26.86	90.27	2013	2015	0.7	Low	Low	10	High
Wangchuk-2016[48]	Bhutan	Trongsa	27.50	90.51	2013	2015	0.5	Low	Low	10	High
Wangchuk-2016[48]	Bhutan	Wangdiphodrang	27.49	89.90	2013	2015	0.5	Low	Low	10	High
Zuluaga-Idarraga-2016[49]	Colombia	Turbo	8.10	-76.73	2012	2014	6.0	Seasonal; API 65 in 2007 and 3 in 2014	Moderate	2	Low
Abreha-2017[12]	Ethiopia	Bishoftu	8.73	39.01	2012	2016	1.4	Not available	Low	7	Low
Abreha-2017[12]	Ethiopia	Batu	6.67	39.42	2012	2016	0.9	Not available	Low	7	Low
Chu-2017a	Thailand	Mae Sot	16.72	98.58	2010	2012	4.0	Not stated	High	10	High
Chu-2017b	Thailand	Mae Sot	16.72	98.58	2012	2014	4.0	Not stated	High	10	High
Grigg-2017	Malaysia	Kudat	6.89	116.85	2013	2016	3.8	Not available	Moderate	10	High
Siqueira-2017[50]	Brazil	Manaus	-3.12	-60.02	2012	2013	6.9	Not stated	High	3	Low

API – annual parasite index; EIR – entomological inoculation rate; **P. vivax* parasite rate estimated by Malaria Atlas Project (MAP) for 2010; †Short relapse periodicity ≤47 days

Table S4. Studies targeted for the analysis but not included

First Author	Treatment arms	Sites	Region	Country	Enrolled	Follow up (days)	Randomised	Recruitment period	Treatment arms*	*Pv* patients enrolled	Pv treated with CQ+/-PQ	Female (%)	Age: Mean (SD)	Age: Median (range)	Reason not included
Bergonzoli-2000[174]	4	2	The Americas	Costa Rica	132	180	Yes	1994	CQ3+PQ14; CQ9+PQ9; CQ5+PQ5; CQ1+PQ1	132	132	Not stated	30.5 (-)		Contact details not up to date
Pukrittayakamee-2000[203]	9	1	Asia-Pacific	Thailand	207	28	Yes	1992-1998	CQ2+PQ14; CQ2; PQ14; Qu; Mfq; Halo; AS; Am; SP	207	60	0	25 (9)		No response from investigators
Singh-2000[204]	1	1	Asia-Pacific	India	75	28	No	1998-1999	CQ3+PQ14	75	75	29.3	28 (14)		No response from investigators
Taylor-2000[166]	2	1	Asia-Pacific	Vietnam	54	28	No	1995	CQ3; Halo	54	29	51.7	Not stated	14 (5-40)	Data not available
Villalobos-Salcedo-2000[205]	2	1	The Americas	Brazil	79	28	Yes	1998	CQ3+PQ14; CQ5+PQ5	79	79	21.5	31.7 (-)		No response from investigators
Abdon-2001[175]	3	1	The Americas	Brazil	120	180	Yes	1994-1995	CQ3+PQ14; CQ1+PQ7; CQ1+PQ5	120	120	37.5	27.3 (-)		Contact details not up to date
Adak-2001[167]	3	1	Asia-Pacific	India	663	365	Yes	Not stated	CQ3; CQ3+PQ5; CQ3+BQ5	663	444	Not stated	Not stated		Data not available
Buchachart-2001[206]	1	1	Asia-Pacific	Thailand	593	28	No	1992-1997	CQ3+PQ14	593	593	37.1	25 (-)		No response from investigators
Dua-2001[207]	1	4	Asia-Pacific	India	5541	540	No	1987-2000	CQ2+PQ5	5541	5541	Not stated	Not stated		No response from investigators
Duarte-2001[208]	1	1	The Americas	Brazil	50	180	No	1997-1998	CQ3+PQ14	50	50	24	31.8 (12.8)		No response from investigators
Soto-2001[209]	1	2	The Americas	Colombia	28	28	No	Not stated	CQ3	28	28	0	Not stated		No response from investigators
Baird-2002[202]	1	1	The Americas	Guyana	13	28	No	1998	CQ3	13	13	21.9	31 (-)		Initial response but no data provided
Blair-Trujillo-2002[216]	1	1	The Americas	Colombia	33	28	No	2001	CQ3	33	33	Not stated	20 (12)		No response from investigators
Castillo-2002[212]	1	1	The Americas	Colombia	50	28	No	1998-1999	CQ3	50	50	84.1	28 (-)		No response from investigators
Congpuong-2002[213]	1	1	Asia-Pacific	Thailand	26	28	No	2000	CQ3+PQ14	26	26	26.9	39 (-)		No response from investigators
Fryauff-2002[211]	1	1	Asia-Pacific	Indonesia	36	28	No	1998	CQ3	36	36	38.9	14 (-)		No response from investigators
Hamedi-2002[214]	1	1	Asia-Pacific	Iran	40	28	No	1999-2001	CQ3	40	40	12.5	27.2 (9.1)		No response from investigators
Maguire-2002[210]	2	1	Asia-Pacific	Indonesia	73	28	No	Not stated	CQ3	73	73	62.5	32 (-)		No response from investigators
Mohapatra-2002[176]	1	1	Asia-Pacific	India	110	365	No	1998-2000	CQ3+PQ14	110	110	36.4	Not stated		Contact details not up to date
Tjitra-2002[215]	3	1	Asia-Pacific	Indonesia	37	28	Yes	1999	CQ3; CQ3+SP; AS+SP	37	9	33	8.8 (-)		No response from investigators
Yadav-2002[217]	2	1	Asia-Pacific	India	1482	365	Yes	1988-1991	CQ1; CQ1+PQ5	1482	1482	Not stated	Not stated		No response from investigators
da Sliva-2003[177]	8	1	The Americas	Brazil	240	180	Yes	Not stated	CQ1+PQ5; CQ1+PQ7; AS+PQ; AS+PQ; AS+PQ; AS+PQ; AS+PQ; AS+PQ	240	60	23.3	32.9 (-)		Contact details not up to date
Machado-2003[218]	1	1	The Americas	Brazil	30	28	No	Not stated	CQ3+PQ14	30	30	Not stated	Not stated		No response from investigators
Nandy-2003[178]	1	2	Asia-Pacific	India	800	28	No	1998-2001	CQ3	800	800	Not stated	Not stated		Contact details not up to date
Pinto-2003[219]	2	1	The Americas	Brazil	132	28	Yes	1997-1998	CQ1+PQ14; CQ3+PQ14	132	132	37.9	30.7 (-)		No response from investigators
Rajgor-2003[220]	2	1	Asia-Pacific	India	273	180	Yes	1998-2000	CQ3; CQ3+PQ14	273	273	12.1	Not stated		No response from investigators
Ruebush-2003[179]	1	2	The Americas	Peru	242	28	No	1998-2001	CQ3	242	242	40.9	19 (-)		Contact details not up to date
Sumawinata-2003[221]	1	1	Asia-Pacific	Indonesia	29	28	No	1995	CQ3	29	29	Not stated	Not stated	24 (5-40)	No response from investigators
Valibayov-2003[222]	1	1	Asia-Pacific	Azerbaijan	153	28	No	Not stated	CQ3+PQ14	153	153	42.5	30.8 (-)		No response from investigators
Hapuarachchi-2004[223]	1	1	Asia-Pacific	Sri Lanka	42	28	No	2002	CQ3	42	42	0	Not stated		No response from investigators
Kurcer-2004[180]	1	2	Asia-Pacific	Turkey	112	28	No	2002	CQ3	112	112	51.4	Not stated		Contact details not up to date
Leslie-2004[189]	3	1	Asia-Pacific	Pakistan	595	270	Yes	2000	CQ3; CQ3+PQ14; CQ3+PQ14	595	595	50.7	12.9 (-)		Missing minimum data
Vijaykadga-2004[224]	1	3	Asia-Pacific	Thailand	161	28	No	2003	CQ3+PQ14	161	161	19.3	32.3 (-)		No response from investigators
Walsh-2004[182]	5	1	Asia-Pacific	Thailand	80	168	Yes	1998-1999	CQ3+TQ7; CQ3+TQ3; CQ3+TQ1; CQ3; CQ3+PQ14	80	25	68	Not stated		Contact details not up to date

Dunne-2005[183]	2	2	Asia-Pacific	India	96	28	Yes	1998-2001	CQ3; Az; CQ3+Az	96	16	6	31.8 (-)		Contact details not up to date
Genton-2005[196]	2	1	Asia-Pacific	Papua New Guinea	18	28	No	1994-1995	CQ3; AQ; Qu+SP	18	5	52	5.1 (-)		Initial response but no data provided
Alvarez-2006[190]	3	2	The Americas	Colombia	210	180	Yes	2001	CQ3+PQ3; CQ3+PQ7; CQ3+PQ14	210	210	33	30.1 (12.8)		Missing minimum data
Krudsood-2006[184]	2	1	Asia-Pacific	Thailand	141	28	Yes	2004-2005	CQ3+PQ7; CQ3+BQ7	141	141	74.6	25.0 (6.7)		Contact details not up to date
Kurcer-2006[181]	1	2	Asia-Pacific	Turkey	91	28	No	2004	CQ3	91	91	34.1	Not stated		Contact details not up to date
Maguire-2006[225]	2	1	Asia-Pacific	Indonesia	243	28	Yes	1996-1999	CQ3; Mfq	243	122	35.2	22.6 (-)		No response from investigators
Tasanor-2006[226]	2	1	Asia-Pacific	Thailand	58	28	Yes	2002-2004	CQ3+PQ14; Qu+PQ14	62	31	41.9	Not stated	22	No response from investigators
de Santana Filho-2007[185]	1	1	The Americas	Brazil	166	28	No	2004-2005	CQ3	166	166	Not stated	Not stated		Contact details not up to date
Kolaczinski-2007[197]	2	1	Asia-Pacific	Afghanistan	190	42	Yes	2004	CQ3; SP+AS	190	96	43	Not stated	8.5	Initial response but no data provided
Krudsood-2007[227]	2	1	Asia-Pacific	Thailand	98	28	Yes	2004-2005	CQ3; AL	98	51	31.4	24.3 (6.3)		No response from investigators
Nateghpour-2007[228]	1	4	Asia-Pacific	Iran	225	28	No	2004-2005	CQ3	225	225	25.6	Not stated		No response from investigators
Osorio-2007[229]	1	1	The Americas	Colombia	22	28	No	2002-2003	CQ3	22	22	50	Not stated		No response from investigators
Barnadas-2008a[198]	1	3	Africa	Madagascar	105	28	No	2006	CQ3	105	105	53	11.2 (-)		Initial response but no data provided
Carmona-Fonseca-2008[191]	1	2	The Americas	Colombia	82	30	Yes	2004	CQ3; CQ3	82	82	62	Not stated		Missing minimum data
Perez-2008[230]	1	1	The Americas	Colombia	50	28	No	2006	CQ3	50	50	42.4	Not stated		No response from investigators
Srivastava-2008[231]	1	2	Asia-Pacific	India	138	28	No	2003-2005	CQ3	138	138	47.1	Not stated		No response from investigators
Teka-2008[199]	1	1	Africa	Ethiopia	87	28	No	2006	CQ3	87	87	41.4	Not stated	16 (0.7-52)	Initial response but no data provided
Carmona-Fonseca-2009[169]	4	2	The Americas	Colombia	188	120	Yes	2001-2003	CQ3+PQ14; CQ3+PQ3; CQ3+PQ3; CQ3+PQ3	188	188	30.4	Not stated		Data not available
Lee-2009b[186]	1	1	Asia-Pacific	Republic of Korea	142	28	No	2007	CQ3+PQ14	142	142	0	Not stated	21 (19-50)	Contact details not up to date
Liang-2009[187]	2	1	Asia-Pacific	Myanmar	48	28	Yes	Not stated	CQ3; CQ3	48	48	Not stated	Not stated		Contact details not up to date
Nateghpour-2009[232]	1	1	Asia-Pacific	Iran	Not available	28	No	Not stated	CQ3	Not available	Not available	Not available	Not available		No response from investigators
Orjuela-Sanchez-2009[192]	2	1	The Americas	Brazil	164	336	No	2004-2007	CQ3+PQ7; CQ3+PQ7	164	164	Not stated	Not stated		Missing minimum data
Rogers-2009[188]	1	1	Asia-Pacific	Cambodia	110	42	No	2006-2008	CQ3	110	110	11	21.2 (6.7)		Contact details not up to date
Carmona-Fonseca-2010[168]	2	1	The Americas	Colombia	79	120	Yes	2005-2008	CQ3+PQ3; CQ3+PQ7	79	79	Not stated	Not stated		Data not available
Daneshvar-2010[193]	1	1	Asia-Pacific	Malaysia	23	28	No	-2007	CQ3+PQ14	23	23	0	38.5 (7.6)		Missing minimum data
Dilmec-2010[233]	1	1	Asia-Pacific	Turkey	42	28	No	Not stated	CQ3	42	42	47.2	Not stated		No response from investigators
Kinzer-2010[234]	1	1	Asia-Pacific	Vanuatu	21	28	No	2005	CQ3	21	21	66.7	Not stated	11 (5-53)	No response from investigators
Takeuchi-2010[235]	2	1	Asia-Pacific	Thailand	216	90	Yes	2007-2009	CQ3+PQ14; CQ3+PQ14	216	216	39.8	Not stated		No response from investigators
Yeshiwondim-2010[236]	2	2	Africa	Ethiopia	290	28	Yes	2003	CQ3; CQ3+PQ14	290	290	45.9	23 (12.0)		No response from investigators
Asih-2011[237]	1	1	Asia-Pacific	Indonesia	73	28	No	2007	CQ3	73	73	50.7	Not stated	13.2 (2-60)	No response from investigators
Congpuong-2011[238]	1	4	Asia-Pacific	Thailand	212	28	No	2009-2010	CQ3	212	212	27.8	Not stated	25 (2-80)	No response from investigators
Ketema-2011[200]	1	1	Africa	Ethiopia	87	28	No	2009	CQ3	87	87	57.4	Not stated	8 (9m-52)	Initial response but no data provided
Maneeboonyang-2011[239]	2	1	Asia-Pacific	Thailand	92	90	Yes	2005-2006	CQ3+PQ14; CQ3+PQ14	92	92	40	Not stated		No response from investigators
Muhamad-2011[240]	1	1	Asia-Pacific	Thailand	130	42	No	2008-2009	CQ3+PQ14	130	130	50.8	Not stated	22	No response from investigators
Van Den Eede-2011[241]	1	1	The Americas	Peru	51	365	No	2008	CQ3+PQ7	51	51	49	Not stated	15 (2-80)	No response from investigators

Yohannes-2011[242]	2	2	Africa	Ethiopia	159	28	No	2004-2005	CQ3; AL	159	71	52.1	Not stated	19 (10-26)	No response from investigators
Anez-2012[201]	1	3	The Americas	Bolivia	223	28	No	2006-2007	CQ3	223	223	42.2	Not stated		Initial response but no data provided
Graf-2012[243]	3	1	The Americas	Peru	540	210	Yes	2005-2008	CQ3+PQ5; CQ3+PQ7; CQ3+PQ14	540	540	Not stated	Not stated		No response from investigators
Pedro-2012[244]	1	1	The Americas	Brazil	47	28	No	2005-2011	CQ3+PQ	47	47	24.5	Not stated		No response from investigators
Saravu-2012[170]	1	1	Asia-Pacific	India	110	28	No	2007-2009	CQ3+PQ14	110	110	26.3	Not stated	28.5	Data not available
Ganguly-2013[245]	2	1	Asia-Pacific	India	250	42	Yes	2011-2012	CQ3; CQ3+PQ14	250	250	10.8	25.2 (-)		No response from investigators
Leang-2013[246]	1	4	Asia-Pacific	Cambodia	390	28	No	2008-2011	CQ3; DP	390	217	29	18.8 (-)		No response from investigators
Liu-2013[247]	2	1	Asia-Pacific	China	255	365	Yes	2009-2010	CQ3+PQ8; ART+NQ	260	132	14	Not stated		No response from investigators
Macareo-2013[248]	2	1	Asia-Pacific	Thailand	20	90	Yes	Not stated	CQ5+PQ14; CQ5+TND	20	6	Not stated	Not stated		No response from investigators
Manandhar-2013[194]	1	4	Asia-Pacific	Nepal	137	180	No	2010-2011	CQ3	137	137	19	Not stated		Missing minimum data
Miahipour-2013[249]	2	2	Asia-Pacific	Iran	180	540	No	2008-2011	CQ3; CQ3+PQ8w	163	163	23.3	Not stated		No response from investigators
Rios-2013[250]	1	1	The Americas	Colombia	152	28	No	2002-2011	CQ3	152	152	39.5	38 (-)		No response from investigators
Zhu-2013[251]	1	1	Asia-Pacific	China	39	28	No	2008-2009	CQ3+PQ8	39	39	42.1	43 (-)		No response from investigators
Amaratunga-2014[252]	1	1	Asia-Pacific	Cambodia	87	28	No	2012-2013	CQ3	87	87	20.7	Not stated	26 (4-68)	No response from investigators
Delgado-Ratto-2014[253]	1	1	The Americas	Peru	37	720	No	2008	CQ3+PQ7	37	37	48.6	Not stated	15	No response from investigators
Liu-2014[254]	1	1	Asia-Pacific	China	750	28	No	2008-2013	CQ3	750	750	41.5	25.2 (6.8)		No response from investigators
Llanos-Cuentas-2014[171]	6	4	Multicentred	Brazil	329	180	Yes	2011-2013	CQ3; CQ3+PQ14; CQ3+TQ; CQ3+TQ; CQ3+TQ; CQ3+TQ	329	104	28.8	34.8 (-)		Data not available
Rajgor-2014[255]		1	Asia-Pacific	India	1556	180	Yes	Not stated	CQ3; CQ3+PQ7; CQ3+PQ14; CQ3+PQ14	1556	1556	4.8	31.2 (-)		No response from investigators
Shalini-2014[195]	1	1	Asia-Pacific	India	125	28	No	2010	CQ3	125	125	5	25.9 (10.5)		Missing minimum data
Ould Ahmedou Salem-2015[256]	1	2	Africa	Mauritania	128	28	No	2013	CQ3	128	128	53.1	27.2 (-)		No response from investigators
Assefa-2015[257]	1	1	Africa	Ethiopia	63	28	No	2014	CQ3	63	63	41.7	Not stated	23 (4-59)	No response from investigators
Cheoymang-2015[258]	1	1	Asia-Pacific	Thailand	85	42	No	2008-2009	CQ3+PQ14	85	85	34.1	Not stated		No response from investigators
Pareek-2015[259]	3	8	Asia-Pacific	India	358	180	Yes	Not stated	CQ3+PQ7; CQ3+PQ14; CQ3+PQ14	358	358	17.3	Not stated	20	No response from investigators
Yuan-2015[260]	1	1	Asia-Pacific	Myanmar	587	42	No	2012-2013	CQ3+PQ8	587	587	46.85	Not stated	9 (1-77)	No response from investigators
Beyene-2016[261]	1	1	Africa	Ethiopia	76	28	No	2014	CQ3	76	76	32	Not stated	19 (3-54)	No response from investigators
Lo-2016[262]	1	1	Asia-Pacific	Myanmar	130	90	No	2011-2013	CQ3	130	130	Not stated	Not stated		No response from investigators
Longley-2016[263]	1	1	Asia-Pacific	Thailand	57	270	No	2014-2015	CQ3+PQ14	57	57	37	Not stated	22 (7-71)	No response from investigators
Mishra-2016[172]	1	5	Asia-Pacific	India	401	42	No	2011-2012	CQ3	401	401	17.5	Not stated		Data not available
Negreiros-2016[264]	1	1	The Americas	Brazil	119	168	No	2014	CQ3+PQ7	119	119	45.4	Not stated	23.4 (5-67.3)	No response from investigators
Valecha-2016[173]	2	9	Asia-Pacific	India	317	42	Yes	2011-2013	CQ3+PQ14; ATM+PIP+PQ14	317	158	8.2	33.7 (13.5)		Data not available
Waqar-2016[265]	1	1	Asia-Pacific	Pakistan	52	28	No	2013	CQ3	52	52	Not stated	5.4 (3.4)		No response from investigators
Seifu-2017[266]	1	1	Africa	Ethiopia	87	28	No	2013	CQ3	87	87	29.7	Not stated	20 (1-65)	No response from investigators

Pv – *P. vivax*; SD – standard deviation; CQ – chloroquine; PQ – primaquine; Qu – Quinine; Mfq – mefloquine; Halo – halofantrine; AS – artesunate; Am - artemether; SP – sulfadoxine-pyrimethamine; BQ – bulaquine; TQ – tafenoquine; Az – azithromycin; AL – artemether-lumefantrine; DP – dihydroartemisinin-piperaquine; ART - artemisinin; NQ - naphthoquine; TND – tinidazole; PQ8w – primaquine weekly for 8 weeks; ATM – arterolane maleate; PIP – piperaquine; [*]Treatment arms in study described as drug and number of days given if CQ+/-PQ.

Table S5. Comparison of baseline characteristics between included and targeted studies

Characteristic	Included studies (n=37)	Targeted studies* (n=101)
Region		
Asia-Pacific, studies (%)	26 (70·3%)	64 (63·4%)
Africa, studies (%)	4 (10·8%)	9 (8·9%)
The Americas, studies (%)	7 (18·9%)	28 (27·7%)
Year of enrolment†		
Pre-2009, studies (%)	15 (40·5%)	66 (74·2%)‡
2009-2017, studies (%)	22 (59·5%)	23 (25·8%)‡
Age, median (IQR)	20·0 (10·0, 31·0)	25·9 (24·3, 31·2)§
Female, % of patients	34·1%	35·2%¶

*Age and female percentage of targeted studies frequency weighted according to number of patients treated with CQ alone or CQ and PQ; †Year of enrolment defined as the year study enrolment completed; ‡Year of enrolment not available from 12 studies; §Mean age not available from 59 studies; ¶Percentage based on frequency weighted median of targeted studies, percentage not available from 17 studies

Table S6A. Total doses of chloroquine by baseline characteristic in patients receiving chloroquine alone

	Chloroquine dose, mg/kg			CQ dose <25 mg/kg
	Number	Median (IQR)	Range	Number (%)
Overall	2990	25·4 (24·2, 28·1)	(6·8, 75·0)	1041 (34·8%)
Gender				
Female	1104	25·9 (24·6, 28·3)	(12·5, 62·5)	307 (27·8%)
Male	1886	25·0 (23·8, 28·1)	(6·8, 75·0)	734 (38·9%)
Age category, years				
<5	359	26·8 (25·0, 28·8)	(6·8, 47·7)	50 (13·9%)
5 to <15	916	26·5 (25·0, 30·0)	(17·5, 75·0)	143 (15·6%)
≥15	1715	25·0 (23·1, 27·1)	(9·4, 44·1)	848 (49·4%)
Weight category, kg				
5 to <15	342	26·8 (25·0, 28·8)	(6·8, 75·0)	54 (15·8%)
15 to <25	574	26·6 (25·0, 30·0)	(17·5, 52·9)	71 (12·4%)
25 to <35	235	26·5 (25·0, 31·0)	(19·5, 46·2)	40 (17·0%)
35 to <45	320	26·4 (25·0, 30·0)	(18·8, 42·9)	82 (25·6%)
45 to <55	649	25·5 (24·5, 28·8)	(12·5, 36·0)	234 (36·1%)
55 to <80	777	24·2 (21·7, 25·0)	(9·4, 32·7)	475 (61·1%)
≥80	93	17·6 (16·3, 18·5)	(13·5, 25·2)	85 (91·4%)
Relapse Periodicity				
Long	1914	26·0 (23·4, 30·0)	(6·8, 75·0)	606 (31·7%)
Short	1076	25·0 (24·3, 26·2)	(17·4, 43·5)	435 (40·4%)
Geographical region				
Asia-Pacific	2112	25·5 (24·3, 27·8)	(6·8, 75·0)	713 (33·8%)
The Americas	289	23·8 (19·6, 25·0)	(13·5, 36·0)	165 (57·1%)
Africa	589	27·3 (24·2, 30·8)	(16·4, 62·5)	163 (27·7%)

IQR = Interquartile range

Table S6B. Total doses of chloroquine and primaquine by baseline characteristic in patients receiving chloroquine and early primaquine

	Chloroquine dose, mg/kg			Chloroquine dose <25 mg/kg	Primaquine dose, mg/kg		
	**Number**	**Median (IQR)**	**Range**	**Number (%)**	**Number**	**Median (IQR)**	**Range**
Overall	1790	25·0 (23·3, 27·3)	(12·6, 75·0)	830 (46·4%)	1789	4·7 (3·4, 6·7)	(0·3, 13·1)
Gender							
Female	571	25·4 (24·3, 28·6)	(13·6, 52·2)	224 (39·2%)	570	4·9 (3·8, 6·8)	(0·4, 9·1)
Male	1219	25·0 (22·7, 26·8)	(12·6, 75·0)	606 (49·7%)	1219	4·3 (3·3, 6·7)	(0·3, 13·1)
Age category, years							
<5	88	27·2 (25·0, 31·2)	(18·8, 45·0)	21 (23·9%)	87	5·1 (4·7, 6·9)	(2·6, 10·5)
5 to <15	413	26·1 (24·8, 30·0)	(13·8, 75·0)	111 (26·9%)	413	5·3 (4·5, 7·0)	(0·3, 13·1)
≥15	1289	24·8 (22·2, 26·3)	(12·6, 44·4)	698 (54·2%)	1289	4·0 (3·2, 6·6)	(0·3, 10·5)
Weight category, kg							
5 to <15	101	28·8 (25·9, 32·6)	(18·8, 75·0)	20 (19·8%)	100	5·1 (4·6, 6·9)	(2·6, 13·1)
15 to <25	208	26·1 (25·0, 30·0)	(20·8, 52·2)	41 (19·7%)	208	5·1 (4·7, 6·6)	(1·4, 9·1)
25 to <35	120	26·8 (25·0, 32·1)	(20·7, 44·4)	30 (25·0%)	120	6·2 (4·5, 7·2)	(2·2, 8·4)
35 to <45	185	25·7 (24·7, 30·0)	(21·1, 38·8)	71 (38·4%)	185	5·2 (4·8, 7·2)	(2·0, 10·5)
45 to <55	413	25·6 (24·8, 28·9)	(13·9, 34·4)	126 (30·5%)	413	5·0 (4·2, 6·9)	(0·3, 8·9)
55 to <80	656	24·2 (21·4, 25·2)	(13·8, 33·2)	435 (66·3%)	656	3·5 (3·1, 4·0)	(0·3, 8·0)
≥80	107	17·5 (16·3, 18·3)	(12·6, 24·5)	107 (100·0%)	107	2·8 (2·5, 3·0)	(1·8, 7·5)
Relapse Periodicity							
Long	902	23·4 (20·5, 27·3)	(12·6, 75·0)	535 (59·3%)	902	3·4 (3·0, 4·0)	(0·3, 13·1)
Short	888	25·4 (24·7, 27·7)	(15·4, 45·0)	295 (33·2%)	887	6·7 (5·0, 7·1)	(0·4, 12·1)
Geographical region							
Asia-Pacific	1203	25·4 (24·6, 28·3)	(12·9, 75·0)	428 (35·6%)	1202	5·7 (4·4, 7·0)	(0·4, 13·1)
The Americas	487	23·1 (19·9, 26·0)	(12·6, 45·0)	321 (65·9%)	487	3·3 (3·0, 3·8)	(2·0, 7·0)
Africa	100	22·7 (21·5, 24·0)	(17·9, 30·0)	81 (81·0%)	100	3·5 (2·6, 4·0)	(0·3, 5·2)

IQR = Interquartile range

Table S7. Risk factors for vomiting in patients treated with chloroquine alone

	Univariate analysis			Multivariate analysis	
	**Number vomited (%)**	**Odds Ratio (95% CI)**	**p value**	**Adjusted Odds Ratio (95% CI)**	**p value**
Chloroquine dose, every mg/kg increase	20/557 (3·6%)	1·19 (1·02, 1·39)	0·0292	1·14 (0·96, 1·34)	0·1270
Age, per every 1 year increase	20/557 (3·6%)	0·92 (0·86, 0·98)	0·0137	0·92 (0·86, 0·98)	0·0142
Gender					
Male	14/369 (3·8%)	Reference	..	Reference	..
Female	6/188 (3·2%)	0·84 (0·31, 2·27)	0·7247	0·59 (0·21, 1·66)	0·3147
Baseline parasitaemia, parasites per μL every ten-times increase	20/557 (3·6%)	1·18 (0·49, 2·85)	0·7106	1·00 (0·51, 1·96)	0·9912
Presence of fever at baseline*					
No	11/343 (3·2%)	Reference	..	Reference	..
Yes	9/201 (4·5%)	1·41 (0·56, 3·54)	0·4588	1·52 (0·69, 3·35)	0·2942
Relapse periodicity					
Long	11/297 (3·7%)	Ref	..	Ref	..
Short	9/260 (3·5%)	0·93 (0·20, 4·28)	0·9281	0·58 (0·15, 2·18)	0·4172

CI = Confidence Interval: *Presence of fever at baseline was only available for 544 of 557 patients

Table S8: Multivariable models for rate of *P. vivax* recurrence between day 7 and 42 in patients receiving chloroquine alone aged less than five years, five to less than 15 years or greater than or equal to 15 years

	Age 0 to <5 years			Age 5 to <15 years			Age ≥15 years		
	Total N (n)*	Adjusted HR (95% CI)	p value	Total N (n)*	Adjusted HR (95% CI)	p value	Total N (n)*	Adjusted HR (95% CI)	p value
Chloroquine dose, every 5 mg/kg increase	359 (100)	0·59 (0·41, 0·86)	0·0058	916 (182)	0·83 (0·62, 1·11)	0·2098	1715 (223)	0·99 (0·76, 1·30)	0·9547
Age, per every 1 year increase	359 (100)	0·89 (0·73, 1·08)	0·2234	916 (182)	0·96 (0·91, 1·01)	0·1549	1715 (223)	0·97 (0·95, 0·98)	<0·0001
Gender									
Male	202 (52)	Reference	..	484 (97)	Reference	..	1200 (167)	Reference	..
Female	157 (48)	1·09 (0·73, 1·64)	0·6690	432 (85)	1·14 (0·84, 1·54)	0·4091	515 (56)	0·77 (0·56, 1·05)	0·1006
Parasitaemia, parasites per µL every ten-times increase	359 (100)	1·13 (0·77, 1·63)	0·5364	916 (182)	1·32 (0·98, 1·79)	0·0675	1715 (223)	1·23 (0·97, 1·56)	0·0892
Relapse periodicity									
Long	197 (28)	Reference	..	630 (52)	Reference	..	1087 (64)	Reference	..
Short	162 (72)	6·98 (2·73, 17·83)	<0·0001	286 (130)	15·10 (5·23, 43·57)	<0·0001	628 (159)	34·67 (10·72, 112·07)	<0·0001

HR – hazard ratio. CI = Confidence Interval *Number of patients (number with recurrence by day 42). The assumption of proportional hazards held for age less than five years (p=0·88) and age five to less than 15 years (p=0·09) but not age greater than or equal to 15 years (p=0· 02) according to the global test. Although the global test held for age five to less than 15 years the test for chloroquine dose did not (p=0.02). For models of age five to less than 15 years and age greater than or equal to 15 years the AHR of chloroquine dose varied with time with a higher hazard ratio in the early follow up to day 21 when recrudescence is more likely (AHR 1·14, 95%CI 0·79-1·63, p=0·4929 and AHR 1·30, 95%CI 0·91-1·87, p=0·1547, respectively) compared to later follow up after day 21 (AHR 0·66, 95%CI 0·45-0·96, p=0·030 and AHR 0·83, 95%CI 0·61-1·15, p=0·266, respectively). To examine the robustness of the parameter estimates, a sensitivity analysis was carried out by removing one study site at a time which showed that the overall coefficient of variation of parameter estimates in the multivariable models was small (all CV <10%; Table S17-19).

Table S9: Risk factors for patent parasitaemia on day 1 in patients receiving chloroquine alone

		Univariable analysis		Multivariable analysis	
	**Total N (n)***	**Crude OR (95% CI)**	**p value**	**Adjusted OR (95% CI)**	**p value**
Chloroquine total dose <25 mg/kg					
Yes	874 (583)	2·09 (1·24, 3·51)	0·0056	1·65 (0·98, 2·78)	0·0595
No	1196 (586)	Ref	..	Ref	..
Age category, years					
≥15	1219 (777)	Ref	..	Ref	..
<5	226 (120)	0·64 (0·33, 1·26)	0·1992	0·78 (0·40, 1·52)	0·4591
5 to <15	625 (272)	0·44 (0·26, 0·75)	0·0023	0·55 (0·34, 0·89)	0·0152
Gender					
Male	1304 (782)	Ref	..	Ref	..
Female	766 (387)	0·68 (0·50, 0·93)	0·0142	0·82 (0·61, 1·10)	0·1793
Baseline parasitaemia, parasites per µL every ten times increase	2070 (1169)	2·35 (1·25, 4·42)	0·0079	2·36 (1·31, 4·26)	0·0042
Relapse periodicity					
Long	1385 (735)	Ref	..	Ref	..
Short	685 (434)	1·53 (0·55, 4·28)	0·4188	1·20 (0·40, 3·56)	0·7473

OR – odds ratio; CI = Confidence Interval *Number of patients (number with positive parasitaemia). Multivariable analysis. Chloroquine was categorised as the relationship between chloroquine dose and the log odds of parasite positivity was not linear.

Table S10. Multivariable model evaluating the relationship between day of parasite clearance and rate of *P. vivax* recurrence between day 7 and 28 in patients receiving chloroquine alone

	**Total N (n)***	**AHR (95% CI)**	**p value**
Day parasites cleared			
Day 1	893 (33)	Ref	..
Day 2	748 (52)	1·38 (0·86, 2·20)	0·1796
Day 3 or after	510 (84)	3·57 (2·09, 6·11)	<0·0001
Chloroquine dose, every 5 mg/kg increase	2151 (169)	0·88 (0·69, 1·12)	0·3150
Age, per every 1 year increase	2151 (169)	0·96 (0·95, 0·98)	<0·0001
Gender			
Male	1352 (114)	Ref	..
Female	799 (55)	0·96 (0·68, 1·35)	0·8102
Parasitaemia, parasites per µL every ten-times increase	2151 (169)	1·17 (0·87, 1·57)	0·2871
Relapse periodicity			
Long	1440 (45)	Ref	..
Short	711 (124)	16·99 (6·38, 45·26)	<0·0001

AHR – adjusted hazard ratio; CI = Confidence Interval; *Number of patients (number with positive parasitaemia). The assumption of proportional hazards did not hold for the model (p<0·0001 according to the global test), with p<0·0001 for chloroquine dose. Similar to the overall model for chloroquine alone, there was an interaction between age and chloroquine dose, and a time varying hazard for patients aged five to less than 15 years or greater than or equal to 15 years. For models of age five to less than 15 years and age greater than or equal to 15 years the AHR of chloroquine dose varied with time with a higher hazard ratio in the early follow up to day 21 when recrudescence is more likely (AHR 1·46, 95% CI 1·01-2·11, p=0·0452 and AHR 1·26, 95% CI 0·85-1·88, p=0·2484, respectively) compared to later follow up after day 21 (AHR 0·59, 95% CI 0·31-1·10, p=0·098 and AHR 0·79, 95% CI 0·46-1·35, p=0·390, respectively).

Table S11. Planned primaquine regimens in patients receiving chloroquine and early primaquine

Total planned dose (mg/kg)	Number of days of regimen	Number (%) (n=1,790)
3·5	7- 9	314 (17·5%)
3·5	14	603 (33·7%)
5·0	10	260 (14·5%)
7	7	170 (9·5%)
7	14	443 (24·8%)

Table S12. Multivariable model for rate of *P. vivax* recurrence between day 7 and 42 in patients receiving chloroquine alone or chloroquine and early primaquine

	Total N (n)*	Adjusted HR (95% CI)	p value
Primaquine			
No	2990 (505)	Ref	..
Yes	1790 (31)	0·10 (0·05, 0·17)	<0·0001
Chloroquine dose, every 5 mg/kg increase	4780 (536)	0·79 (0·67, 0·93)	0·0042
Age, per every 1 year increase	4780 (536)	0·97 (0·96, 0·97)	<0·0001
Gender			
Male	3105 (340)	Ref	..
Female	1675 (196)	0·93 (0·78, 1·12)	0·4661
Parasitaemia, parasites per µL every ten-times increase	4780 (536)	1·28 (1·09, 1·50)	0·0022
Relapse periodicity			
Long	2816 (155)	Ref	..
Short	1964 (381)	13·63 (5·54, 33·56)	<0·0001

HR – hazard ratio. CI = Confidence Interval *Number of patients (number with recrudescence by day 42). The assumption of proportional hazards held for the model (p=0·06 according to the global test), although p=0·002 for chloroquine dose. Similar to the overall model for chloroquine alone, there was an interaction between age and chloroquine dose, and a time varying hazard for patients aged five to less than 15 years or greater than or equal to 15 years. For models of age five to less than 15 years and age greater than or equal to 15 years the AHR of chloroquine dose varied with time with a higher hazard ratio in the early follow up to day 21 when recrudescence is more likely (AHR 1·11, 95% CI 0·77-1·59, p=0·5817 and AHR 1·42, 95% CI 1·02-1·97, p=0·0378, respectively) compared to later follow up after day 21 (AHR 0·69, 95% CI 0·48-0·98, p=0·040 and AHR 0·80, 95% CI 0·59-1·07, p=0·127, respectively). To examine the robustness of the parameter estimates, a sensitivity analysis was carried out by removing one study site at a time which showed that the overall coefficient of variation of parameter estimates in the multivariable model was small (all CV <7%; Table S20).

Table S13. Rate of *P. vivax* recurrence between day 7 and 42 in the subgroup of patients receiving chloroquine and early primaquine

	**Total N (n)***	**Adjusted HR (95% CI)**	**p value**
Primaquine total dose <7 mg/kg†			
No	1453 (27)	Ref	..
Yes	336 (4)	1·88 (0·54, 6·51)	0·3183
Chloroquine dose, every 5 mg/kg increase	1790 (31)	0·72 (0·40, 1·27)	0·2514
Age, per every 1 year increase	1790 (31)	0·99 (0·97, 1·02)	0·6636
Gender			
Male	1219 (24)	Ref	..
Female	571 (7)	0·52 (0·22, 1·22)	0·1302
Parasitaemia, parasites per μL every ten-times increase	1790 (31)	1·53 (0·81, 2·86)	0·1882
Relapse periodicity			
Long	902 (11)	Ref	..
Short	888 (20)	3·16 (0·54, 18·49)	0·2011

HR – hazard ratio. CI = Confidence Interval *Number of patients (number with recrudescence by day 42). †Primaquine dose did not have a linear association with risk of recurrence. The assumption of proportional hazards held for the model (p=0·17 according to the global test).

Table S14. Multivariable model for rate of *P. vivax* recurrence between day 7 and 42 in patients aged less than five years receiving chloroquine alone in long relapse periodicity regions

	Total N (n)*	Adjusted HR (95% CI)	p value
Chloroquine dose, every 5 mg/kg increase	197 (28)	0·63 (0·42, 0·96)	0·0314
Age, every 1 year increase	197 (28)	0·77 (0·55, 1·10)	0·1513
Gender			
Male	123 (20)	Ref	Ref
Female	74 (8)	0·61 (0·26, 1·42)	0·2499
Parasitaemia, parasites per µL every ten-times increase	197 (28)	1·25 (0·58, 2·73)	0·5688

HR – hazard ratio. CI = Confidence Interval *Number of patients (number with recrudescence by day 42). The assumption of proportional hazards held for the model (p=0·90 for the global test)

Table S15. Accuracy of presence of parasite positivity in determining risk of recurrence prior to day 28 in patients receiving chloroquine alone

Parasite presence	Sensitivity	Specificity	PPV	NPV
Day 1	75·9% (110/145)	41·4% (923/2231)	7·8% (110/1418)	96·3% (923/958)
Day 2	38·7% (82/212)	80·6% (2161/2682)	13·6% (82/602)	94·3% (2162/2292)
Day 3	12·3% (32/261)	95·8% (2428/2535)	23·0% (32/139)	91·4% (2428/2657)

PPV = Positive predictive value; NPV = Negative predictive value.

Table S16. Sensitivity analysis for rate of *P. vivax* recurrence between day 7 to 42 for the overall model of patients that received chloroquine alone

Variable	Range of HR	Coefficient of Variation (%)*
Chloroquine dose, per every 5 mg/kg increase	0·79-0·86	1·37
Age, per every 1 year increase	0·96-0·97	0·07
Gender		
Male	1	..
Female	0·93-1·00	0·91
Parasitaemia, parasites per μL every ten-times increase	1·17-1·33	1·33
Relapse periodicity		
Long	1	..
Short	17·31-25·19	5·77

Sensitivity analysis was generated by removing each study site one at a time

*The coefficient of variation calculated as standard deviation divided by the mean of the estimates. There were a total of 66 sites.

Table S17. Sensitivity analysis for the model of rate of *P. vivax* recurrence between day 7 and 42 in patients receiving chloroquine alone aged less than 5 years

Variable	Range of HR	Coefficient of Variation (%)*
Chloroquine dose, per every 5 mg/kg increase	0·53-0·64	2·82
Age, per every 1 year increase	0·86-0·92	0·93
Gender		
Male	1	..
Female	0·99-1·19	2·47
Parasitaemia, parasites per μL every ten-times increase	1·07-1·24	1·62
Relapse periodicity		
Long	1	..
Short	5·28-8·67	6·94

Sensitivity analysis was generated by removing each study site one at a time

*The coefficient of variation calculated as standard deviation divided by the mean of the estimates. There were a total of 37 sites.

Table S18. Sensitivity analysis for the model of rate of *P. vivax* recurrence between day 7 and 42 in patients receiving chloroquine alone aged five to less than 15 years

Variable	Range of HR	Coefficient of Variation (%)*
Chloroquine dose, per every 5 mg/kg increase	0·67-0·87	3·17
Age, per every 1 year increase	0·95-0·97	0·33
Gender		
Male	1	..
Female	1·07-1·19	1·46
Parasitaemia, parasites per μL every ten-times increase	1·25-1·37	1·41
Relapse periodicity		
Long	1	..
Short	12·21-18·11	7·18

Sensitivity analysis was generated by removing each study site one at a time

*The coefficient of variation calculated as standard deviation divided by the mean of the estimates. There were a total of 50 sites.

Table S19. Sensitivity analysis for the model of rate of *P. vivax* recurrence between day 7 and 42 in patients receiving chloroquine alone aged 15 years or more

Variable	Range of HR	Coefficient of Variation (%)*
Chloroquine dose, per every 5 mg/kg increase	0·93-1·07	1·57
Age, per every 1 year increase	0·96-0·97	0·09
Gender		
Male	1	..
Female	0·73-0·87	2·22
Parasitaemia, parasites per µL every ten-times increase	1·03-1·35	2·51
Relapse periodicity		
Long	1	..
Short	29·22-45·34	8·3

Sensitivity analysis was generated by removing each study site one at a time

*The coefficient of variation calculated as standard deviation divided by the mean of the estimates. There were a total of 65 sites.

Table S20. Sensitivity analysis for the model of rate of *P. vivax* recurrence between day 7 and 42 in patients receiving chloroquine alone or chloroquine and early primaquine

Variable	Range of HR	Coefficient of Variation (%)*
Primaquine		
No	1	..
Yes	0·07-0·14	6·74
Chloroquine dose, per every 5 mg/kg increase	0·76-0·82	1·27
Age, per every 1 year increase	0·96-0·97	0·07
Gender		
Male	1	..
Female	0·91-0·96	0·87
Parasitaemia, parasites per µL every ten-times increase	1·19-1·33	1·22
Relapse periodicity		
Long	1	..
Short	11·45-18·52	6·85

Sensitivity analysis was generated by removing each study site one at a time

*The coefficient of variation calculated as standard deviation divided by the mean of the estimates. There were a total of 66 sites.

References of studies not included in analysis

69. Alving AS, Johnson CF, Tarlov AR, Brewer GJ, Kellermeyer RW, Carson PE. Mitigation of the haemolytic effect of primaquine and enhancement of its action against exoerythrocytic forms of the Chesson strain of *Plasmodium vivax* by intermittent regimens of drug administration: a preliminary report. *Bull World Health Organ* 1960; **22**: 621-31.

70. Basavaraj HR. Observations on the treatment of 678 malaria cases with primaquine in an area free from maiaria transmission in Mysore State, India. *Indian J Malariol* 1960; **14**: 269-81.

71. Diwan. Primaquin Trials as Antirelapse (Radical) Treatment for Malaria conducted in National Malaria Eradication Programme Units (East) Meerut and Mathura (Uttar Pradesh). *Bull Nat Soc India Malar Other Mosquito Borne Dis* 1962; **10**(2): 91-102.

72. Gomez Mendoza I, World Health Organization. Comparative study of two regimens of radical treatment of vivax malaria in Mexico. Geneva: World Health Organization; 1965.

73. Comer RD, Young MD, Porter JA, Jr., Gauld JR, Merritt W. Chloroquine resistance in *Plasmodium falciparum* malaria on the Pacific coast of Colombia. *Am J Trop Med Hyg* 1968; **17**(6): 795-9.

74. Fisher GU, Gordon MP, Lobel HO, Runcik K. Malaria in soldiers returning from Vietnam. Epidemiologic, therapeutic, and clinical studies. *Am J Trop Med Hyg* 1970; **19**(1): 27-39.

75. Contacos PG, Coatney GR, Collins WE, Briesch PE, Jeter MH. Five day primaquine therapy--an evaluation of radical curative activity against vivax malaria infection. *Am J Trop Med Hyg* 1973; **22**(6): 693-5.

76. Sharma MID, Sehgal PN, Vaid BK, Dubey RC, Nagendra S, Paithne PK. Effectiveness of drug schedule being followed under the National Malaria Eradication Programme, India, for radical cure of vivax malaria cases. *J Comm Dis* 1973; **5**: 167-74.

77. Contacos PG, Collins WE, Chin W, Jeter MH, Briesch PE. Combined chloroquine-primaquine therapy against vivax malaria. *Am J Trop Med Hyg* 1974; **23**(2): 310-2.

78. Kaplan MH, Bernstein LS. Improved therapy for Vietnam acquired vivax malaria. *Mil Med* 1974; **141**(6): 444-8.

79. Miller LH, Wyler DJ, Glew RH, Collins WE, Contacos PG. Sensitivity of four Central American strains of *Plasmodium vivax* to primaquine. *Am J Trop Med Hyg* 1974; **23**(2): 309-10.

80. Clyde DF, McCarthy VC. Radical cure of Chesson strain vivax malaria in man by 7, not 14, days of treatment with primaquine. *Am J Trop Med Hyg* 1977; **26**(3): 562-3.

81. Roy RG, Chakrapani KP, Dhinagaran D, Sitaraman NL, Ghosh RB. Efficacy of 5-day radical treatment of *P. vivax* infection in Tamil Nadu. *Indian J Med Res* 1977; **65**(5): 652-6.

82. Saint-Yves IF. Comparison of treatment schedules for *Plasmodium vivax* infections in the Solomon Islands. *P N G Med J* 1977; **20**(2): 62-5.

83. Cedillos RA, Warren M, Jeffery GM. Field evaluation of primaquine in the control of *Plasmodium vivax*. *Am J Trop Med Hyg* 1978; **27**(3): 466-72.

84. Roy RG, Shanmugham CA, Chakrapani KP, Ganesan AV. Results of 5-day course of radical treatment of *Plasmodium vivax* in six districts of Tamil Nadu. *Indian J Med Res* 1979; **69**: 939-43.

85. Darlow B, Vrbova H, Gibney S, Jolley D, Stace J, Alpers M. Sulfadoxine-pyrimethamine for the treatment of acute malaria in children of Papua New Guinea. II. *Plasmodium vivax*. *Am J Trop Med Hyg* 1982; **31**(1): 10-3.

86. Lal H. A comparative trial of oral chloroquine and oral co-trimoxazole in vivax malaria in children. *Am J Trop Med Hyg* 1982; **31**(3 Pt 1): 438-40.

87. Appavoo NC, Roy RG, Kapali V. Results of 3-day radical treatment of *Plasmodium vivax* in North Arcot and South Arcot Districts of Tamil Nadu. *Indian J Malariol* 1984; **21**(1): 21-4.

88. Huei BL, Shen SL, Wang KS, Gao SF, Cao SZ. [The radical efficacy of pyronaridine-primaquine combination in tertian malaria]. *Ji Sheng Chong Xue Yu Ji Sheng Chong Bing Za Zhi* 1984; **2**(4): 260-2.

89. Alcantara AK, Uylangco CV, Sangalang RP, Cross JH. A comparative clinical study of mefloquine and chloroquine in the treatment of vivax malaria. *Southeast Asian J Trop Med Public Health* 1985; **16**(4): 534-8.

90. Dixon KE, Pitaktong U, Phintuyothin P. A clinical trial of mefloquine in the treatment of *Plasmodium vivax* malaria. *Am J Trop Med Hyg* 1985; **34**(3): 435-7.

91. Harinasuta T, Bunnag D, Lasserre R, Leimer R, Vinijanont S. Trials of mefloquine in vivax and of mefloquine plus 'fansidar' in falciparum malaria. *Lancet* 1985; **1**(8434): 885-8.

92. Ebisawa I, Ohara H. A combination of sulfamonomethoxine and pyrimethamine versus other drugs for the treatment of malaria. *Jpn J Exp Med* 1986; **56**(5): 213-9.

93. Maisonneuve H, Joly F, John M, Carles G, Rossignol JF. [Efficacy of halofantrine in *Plasmodium falciparum* or *Plasmodium vivax* malaria in a resistance area (French Guiana)]. *Presse Med* 1988; **17**(3): 99-102.

94. Da Silva AR, Silva Cde M, Branco Mdos R, Branco Filho JR. [Results of the use of a therapeutic protocol for *Plasmodium vivax* for 5 days in 3 municipalities of the Sao Luis Island, State of Maranhao, Brazil]. *Rev Soc Bras Med Trop* 1989; **22**(3): 131-6.

95. Sinha S, Dua VK, Sharma VP. Efficacy of 5 day radical treatment of primaquine in *Plasmodium vivax* cases at the BHEL industrial complex, Hardwar (U.P.). *Indian J Malariol* 1989; **26**(2): 83-6.

96. Singh N, Mishra AK, Sharma VP. Radical treatment of vivax malaria in Madhya Pradesh, India. *Indian J Malariol* 1990; **27**(1): 55-6.

97. Bunnag D, Karbwang J, Thanavibul A, et al. High dose of primaquine in primaquine resistant vivax malaria. *Trans R Soc Trop Med Hyg* 1994; **88**(2): 218-9.

98. Pukrittayakamee S, Vanijanonta S, Chantra A, Clemens R, White NJ. Blood stage antimalarial efficacy of primaquine in *Plasmodium vivax* malaria. *J Infect Dis* 1994; **169**(4): 932-5.

99. Prasad RN, Virk KJ, Sharma VP. Relapse/reinfection patterns of *Plasmodium vivax* infection: a four year study. *Southeast Asian J Trop Med Public Health* 1991; **22**(4): 499-503.

100. Jagota SC. Halofantrine in the treatment of acute malaria: a multi-centre study in 268 patients. *Curr Med Res Opin* 1993; **13**(3): 140-4.

101. Nguyen DS, Dao BH, Nguyen PD, et al. Treatment of malaria in Vietnam with oral artemisinin. *Am J Trop Med Hyg* 1993; **48**(3): 398-402.

102. Rao KS, Kamalakar KV. Efficacy and safety of halofantrine in acute malaria. *J Assoc Physicians India* 1993; **41**(8): 507-8.

103. Li GQ, Guo XB, Fu LC, Jian HX, Wang XH. Clinical trials of artemisinin and its derivatives in the treatment of malaria in China. *Trans R Soc Trop Med Hyg* 1994; **88 Suppl 1**: S5-6.

104. Pukrittayakamee S, Viravan C, Charoenlarp P, Yeamput C, Wilson RJ, White NJ. Antimalarial effects of rifampin in *Plasmodium vivax* malaria. *Antimicrob Agents Chemother* 1994; **38**(3): 511-4.

105. Baird JK, Basri H, Subianto B, et al. Treatment of chloroquine-resistant *Plasmodium vivax* with chloroquine and primaquine or halofantrine. *J Infect Dis* 1995; **171**(6): 1678-82.

106. Jelinek T, Nothdurft HD, Von Sonnenburg F, Loscher T. Long-term efficacy of primaquine in the treatment of vivax malaria in nonimmune travelers. *Am J Trop Med Hyg* 1995; **52**(4): 322-4.

107. Potkar CN, Kshirsagar NA, Kathuria R. Resurgence of malaria and drug resistance in *Plasmodium falciparum* and *Plasmodium vivax* species in Bombay. *J Assoc Physicians India* 1995; **43**(5): 336-8.

108. Marlar T, Myat Phone K, Aye Yu S, Khaing Khaing G, Ma S, Myint O. Development of resistance to chloroquine by *Plasmodium vivax* in Myanmar. *Trans R Soc Trop Med Hyg* 1995; **89**(3): 307-8.

109. Baird JK, Sustriayu Nalim MF, Basri H, et al. Survey of resistance to chloroquine by *Plasmodium vivax* in Indonesia. *Trans R Soc Trop Med Hyg* 1996; **90**(4): 409-11.

110. Baird JK, Sismadi P, Masbar S, et al. Chloroquine sensitive *Plasmodium falciparum* and *P. vivax* in central Java, Indonesia. *Trans R Soc Trop Med Hyg* 1996; **90**(4): 412-3.

111. Baird JK, Caneta-Miguel E, Masbar S, et al. Survey of resistance to chloroquine of falciparum and vivax malaria in Palawan, The Philippines. *Trans R Soc Trop Med Hyg* 1996; **90**(4): 413-4.

112. Looareesuwan S, Viravan C, Webster HK, Kyle DE, Hutchinson DB, Canfield CJ. Clinical studies of atovaquone, alone or in combination with other antimalarial drugs, for treatment of acute uncomplicated malaria in Thailand. *Am J Trop Med Hyg* 1996; **54**(1): 62-6.

113. Srivastava HC, Sharma SK, Bhatt RM, Sharma VP. Studies on *Plasmodium vivax* relapse pattern in Kheda district, Gujarat. *Indian J Malariol* 1996; **33**(4): 173-9.

114. Baird JK, Wiady I, Fryauff DJ, et al. In vivo resistance to chloroquine by *Plasmodium vivax* and *Plasmodium falciparum* at Nabire, Irian Jaya, Indonesia. *Am J Trop Med Hyg* 1997; **56**(6): 627-31.

115. Fryauff DJ, Baird JK, Candradikusuma D, et al. Survey of in vivo sensitivity to chloroquine by *Plasmodium falciparum* and *P. vivax* in Lombok, Indonesia. *Am J Trop Med Hyg* 1997; **56**(2): 241-4.

116. Fryauff DJ, Baird JK, Basri H, et al. Halofantrine and primaquine for radical cure of malaria in Irian Jaya, Indonesia. *Ann Trop Med Parasitol* 1997; **91**(1): 7-16.

117. Xu C, Ding Y, Qi Z. [Efficacy of dihydroartemisinin in treatment of 37 malaria cases]. *Zhonghua Nei Ke Za Zhi* 1997; **36**(3): 187-9.

118. Fryauff DJ, Tuti S, Mardi A, et al. Chloroquine-resistant *Plasmodium vivax* in transmigration settlements of West Kalimantan, Indonesia. *Am J Trop Med Hyg* 1998; **59**(4): 513-8.

119. Fryauff DJ, Soekartono, Tuti S, et al. Survey of resistance in vivo to chloroquine of *Plasmodium falciparum* and *P. vivax* in North Sulawesi, Indonesia. *Trans R Soc Trop Med Hyg* 1998; **92**(1): 82-3.

120. Gogtay N, Garg M, Kadam V, Kamtekar K, Kshirsagar NA. A 5 days primaquine regimen as anti-relapse therapy for *Plasmodium vivax*. *Trans R Soc Trop Med Hyg* 1998; **92**(3): 341.

121. Pinto AY, Ventura AM, Calvosa VS, et al. [Clinical efficacy of four schemes for vivax malaria treatment in children]. *J Pediatr (Rio J)* 1998; **74**(3): 222-7.

122. Fryauff DJ, Sumawinata I, Purnomo, et al. In vivo responses to antimalarials by *Plasmodium falciparum* and *Plasmodium vivax* from isolated Gag Island off northwest Irian Jaya, Indonesia. *Am J Trop Med Hyg* 1999; **60**(4): 542-6.

123. Gogtay NJ, Desai S, Kamtekar KD, Kadam VS, Dalvi SS, Kshirsagar NA. Efficacies of 5- and 14-day primaquine regimens in the prevention of relapses in *Plasmodium vivax* infections. *Ann Trop Med Parasitol* 1999; **93**(8): 809-12.

124. Kaneko A, Bergqvist Y, Takechi M, et al. Intrinsic efficacy of proguanil against falciparum and vivax malaria independent of the metabolite cycloguanil. *J Infect Dis* 1999; **179**(4): 974-9.

125. Li X, Li C, Che L, et al. [Observation on efficacy of artemether compound against vivax malaria]. *Zhongguo Ji Sheng Chong Xue Yu Ji Sheng Chong Bing Za Zhi* 1999; **17**(3): 175-7.

126. Lim CS, Kim YK, Lee KN, et al. Response to chloroquine of *Plasmodium vivax* among South Korean soldiers. *Ann Trop Med Parasitol* 1999; **93**(6): 565-8.

127. Looareesuwan S, Wilairatana P, Glanarongran R, et al. Atovaquone and proguanil hydrochloride followed by primaquine for treatment of *Plasmodium vivax* malaria in Thailand. *Trans R Soc Trop Med Hyg* 1999; **93**(6): 637-40.

128. Looareesuwan S, Wilairatana P, Krudsood S, et al. Chloroquine sensitivity of *Plasmodium vivax* in Thailand. *Ann Trop Med Parasitol* 1999; **93**(3): 225-30.

129. Rowland M, Durrani N. Randomized controlled trials of 5- and 14-days primaquine therapy against relapses of vivax malaria in an Afghan refugee settlement in Pakistan. *Trans R Soc Trop Med Hyg* 1999; **93**(6): 641-3.

130. Walsh DS, Looareesuwan S, Wilairatana P, et al. Randomized dose-ranging study of the safety and efficacy of WR 238605 (Tafenoquine) in the prevention of relapse of *Plasmodium vivax* malaria in Thailand. *J Infect Dis* 1999; **180**(4): 1282-7.

131. Wilairatana P, Silachamroon U, Krudsood S, et al. Efficacy of primaquine regimens for primaquine-resistant *Plasmodium vivax* malaria in Thailand. *Am J Trop Med Hyg* 1999; **61**(6): 973-7.

132. Pukrittayakamee S, Clemens R, Chantra A, et al. Therapeutic responses to antibacterial drugs in vivax malaria. *Trans R Soc Trop Med Hyg* 2001; **95**(5): 524-8.

133. Lacy MD, Maguire JD, Barcus MJ, et al. Atovaquone/proguanil therapy for *Plasmodium falciparum* and *Plasmodium vivax* malaria in Indonesians who lack clinical immunity. *Clin Infect Dis* 2002; **35**(9): e92-5.

134. Silachamroon U, Krudsood S, Treeprasertsuk S, et al. Clinical trial of oral artesunate with or without high-dose primaquine for the treatment of vivax malaria in Thailand. *Am J Trop Med Hyg* 2003; **69**(1): 14-8.

135. Hamedi Y, Safa O, Zare S, Tan-ariya P, Kojima S, Looareesuwan S. Therapeutic efficacy of artesunate in *Plasmodium vivax* malaria in Thailand. *Southeast Asian J Trop Med Public Health* 2004; **35**(3): 570-4.

136. Khan MZ, Isani Z, Ahmed TM, et al. Efficacy and safety of halofantrine in Pakistani children and adults with malaria caused by *P. falciparum* and *P. vivax*. *Southeast Asian J Trop Med Public Health* 2006; **37**(4): 613-8.

137. Dao NV, Cuong BT, Ngoa ND, et al. Vivax malaria: preliminary observations following a shorter course of treatment with artesunate plus primaquine. *Trans R Soc Trop Med Hyg* 2007; **101**(6): 534-9.

138. Hasugian AR, Purba HL, Kenangalem E, et al. Dihydroartemisinin-piperaquine versus artesunate-amodiaquine: superior efficacy and posttreatment prophylaxis against multidrug-resistant *Plasmodium falciparum* and *Plasmodium vivax* malaria. *Clin Infect Dis* 2007; **44**(8): 1067-74.

139. Ratcliff A, Siswantoro H, Kenangalem E, et al. Two fixed-dose artemisinin combinations for drug-resistant falciparum and vivax malaria in Papua, Indonesia: an open-label randomised comparison. *Lancet* 2007; **369**(9563): 757-65.

140. Barnadas C, Tichit M, Bouchier C, et al. *Plasmodium vivax dhfr* and *dhps* mutations in isolates from Madagascar and therapeutic response to sulphadoxine-pyrimethamine. *Malar J* 2008; **7**: 35.

141. Krudsood S, Tangpukdee N, Wilairatana P, et al. High-dose primaquine regimens against relapse of *Plasmodium vivax* malaria. *Am J Trop Med Hyg* 2008; **78**(5): 736-40.

142. Hasugian AR, Tjitra E, Ratcliff A, et al. In vivo and in vitro efficacy of amodiaquine monotherapy for treatment of infection by chloroquine-resistant *Plasmodium vivax*. *Antimicrob Agents Chemother* 2009; **53**(3): 1094-9.

143. Congpuong K, Bualombai P, Banmairuroi V, Na-Bangchang K. Compliance with a three-day course of artesunate-mefloquine combination and baseline anti-malarial treatment in an area of Thailand with highly multidrug resistant falciparum malaria. *Malar J* 2010; **9**: 43.

144. Pukrittayakamee S, Imwong M, Chotivanich K, Singhasivanon P, Day NP, White NJ. A comparison of two short-course primaquine regimens for the treatment and radical cure of *Plasmodium vivax* malaria in Thailand. *Am J Trop Med Hyg* 2010; **82**(4): 542-7.

145. Abdallah TM, Ali AA, Bakri M, Gasim GI, Musa IR, Adam I. Efficacy of artemether-lumefantrine as a treatment for uncomplicated *Plasmodium vivax* malaria in eastern Sudan. *Malar J* 2012; **11**: 404.

146. Betuela I, Rosanas-Urgell A, Kiniboro B, et al. Relapses contribute significantly to the risk of *Plasmodium vivax* infection and disease in Papua New Guinean children 1-5 years of age. *J Infect Dis* 2012; **206**(11): 1771-80.

147. Eibach D, Ceron N, Krishnalall K, et al. Therapeutic efficacy of artemether-lumefantrine for *Plasmodium vivax* infections in a prospective study in Guyana. *Malar J* 2012; **11**: 347.

148. Tjitra E, Hasugian AR, Siswantoro H, et al. Efficacy and safety of artemisinin-naphthoquine versus dihydroartemisinin-piperaquine in adult patients with uncomplicated malaria: a multi-centre study in Indonesia. *Malar J* 2012; **11**: 153.

149. Pasaribu AP, Chokejindachai W, Sirivichayakul C, et al. A randomized comparison of dihydroartemisinin-piperaquine and artesunate-amodiaquine combined with primaquine for radical treatment of vivax malaria in Sumatera, Indonesia. *J Infect Dis* 2013; **208**(11): 1906-13.

150. Senn N, Rarau P, Manong D, et al. Effectiveness of artemether/lumefantrine for the treatment of uncomplicated *Plasmodium vivax* and *P. falciparum* malaria in young children in Papua New Guinea. *Clin Infect Dis* 2013; **56**(10): 1413-20.

151. Sutanto I, Tjahjono B, Basri H, et al. Randomized, open-label trial of primaquine against vivax malaria relapse in Indonesia. *Antimicrob Agents Chemother* 2013; **57**(3): 1128-35.

152. Laman M, Moore BR, Benjamin JM, et al. Artemisinin-naphthoquine versus artemether-lumefantrine for uncomplicated malaria in Papua New Guinean children: an open-label randomized trial. *PLoS Med* 2014; **11**(12): e1001773.

153. Lon C, Manning JE, Vanachayangkul P, et al. Efficacy of two versus three-day regimens of dihydroartemisinin-piperaquine for uncomplicated malaria in military personnel in northern Cambodia: an open-label randomized trial. *PLoS One* 2014; **9**(3): e93138.

154. Kheng S, Muth S, Taylor WR, et al. Tolerability and safety of weekly primaquine against relapse of *Plasmodium vivax* in Cambodians with glucose-6-phosphate dehydrogenase deficiency. *BMC Med* 2015; **13**: 203.

155. Nelwan EJ, Ekawati LL, Tjahjono B, et al. Randomized trial of primaquine hypnozoitocidal efficacy when administered with artemisinin-combined blood schizontocides for radical cure of *Plasmodium vivax* in Indonesia. *BMC Med* 2015; **13**: 294.

156. Alecrim MG, Carvalho LM, Fernandes MC, et al. [Malaria treatment with artesunate (retocaps) in children of the Brazilian Amazon]. *Rev Soc Bras Med Trop* 2000; **33**(2): 163-8.

157. Fernandopulle BM, Weeraratne CL, Weerasuriya K, Karunaweera ND. Efficacy of a five-day course of primaquine in preventing relapses in *Plasmodium vivax* malaria--a pilot study. *Ceylon Med J* 2003; **48**(1): 32.

158. Yeramian P, Meshnick SR, Krudsood S, et al. Efficacy of DB289 in Thai patients with *Plasmodium vivax* or acute, uncomplicated *Plasmodium falciparum* infections. *J Infect Dis* 2005; **192**(2): 319-22.

159. Benjamin J, Moore B, Lee ST, et al. Artemisinin-naphthoquine combination therapy for uncomplicated pediatric malaria: a tolerability, safety, and preliminary efficacy study. *Antimicrob Agents Chemother* 2012; **56**(5): 2465-71.

160. Marfurt J, Mueller I, Sie A, et al. Low efficacy of amodiaquine or chloroquine plus sulfadoxine-pyrimethamine against *Plasmodium falciparum* and *P. vivax* malaria in Papua New Guinea. *Am J Trop Med Hyg* 2007; **77**(5): 947-54.

161. Karunajeewa HA, Mueller I, Senn M, et al. A trial of combination antimalarial therapies in children from Papua New Guinea. *N Engl J Med* 2008; **359**(24): 2545-57.

162. Phyo AP, Jittamala P, Nosten FH, et al. Antimalarial activity of artefenomel (OZ439), a novel synthetic antimalarial endoperoxide, in patients with *Plasmodium falciparum* and *Plasmodium vivax* malaria: an open-label phase 2 trial. *Lancet Infect Dis* 2016; **16**(1): 61-9.

163. White NJ, Duong TT, Uthaisin C, et al. Antimalarial Activity of KAF156 in Falciparum and Vivax Malaria. *N Engl J Med* 2016; **375**(12): 1152-60.

164. McGready R, Thwai KL, Cho T, et al. The effects of quinine and chloroquine antimalarial treatments in the first trimester of pregnancy. *Trans R Soc Trop Med Hyg* 2002; **96**(2): 180-4.

165. Moore BR, Benjamin JM, Auyeung SO, et al. Safety, tolerability and pharmacokinetic properties of coadministered azithromycin and piperaquine in pregnant Papua New Guinean women. *Br J Clin Pharmacol* 2016; **82**(1): 199-212.

166. Taylor WR, Doan HN, Nguyen DT, et al. Assessing drug sensitivity of *Plasmodium vivax* to halofantrine or choroquine in southern, central Vietnam using an extended 28-day in vivo test and polymerase chain reaction genotyping. *Am J Trop Med Hyg* 2000; **62**(6): 693-7.

167. Adak T, Valecha N, Sharma VP. *Plasmodium vivax* polymorphism in a clinical drug trial. *Clin Diagn Lab Immunol* 2001; **8**(5): 891-4.

168. Carmona-Fonseca J. Vivax malaria in children: Recurrences with standard total dose of primaquine administered in 3 vs. 7 days *Iatreia* 2010; **23**(1): 10-20.

169. Carmona-Fonseca J, Maestre A. Prevention of *Plasmodium vivax* malaria recurrence: efficacy of the standard total dose of primaquine administered over 3 days. *Acta Trop* 2009; **112**(2): 188-92.

170. Saravu K, Acharya V, Kumar K, Kumar R. *Plasmodium vivax* remains responsive to chloroquine with primaquine treatment regimen: a prospective cohort study from tertiary care teaching hospital in southern India. *Trop Doct* 2012; **42**(3): 163-4.

171. Llanos-Cuentas A, Lacerda MV, Rueangweerayut R, et al. Tafenoquine plus chloroquine for the treatment and relapse prevention of *Plasmodium vivax* malaria (DETECTIVE): a multicentre, double-blind, randomised, phase 2b dose-selection study. *Lancet* 2014; **383**(9922): 1049-58.

172. Mishra N, Srivastava B, Bharti RS, et al. Monitoring the efficacy of antimalarial medicines in India via sentinel sites: Outcomes and risk factors for treatment failure. *J Vector Borne Dis* 2016; **53**(2): 168-78.

173. Valecha N, Savargaonkar D, Srivastava B, et al. Comparison of the safety and efficacy of fixed-dose combination of arterolane maleate and piperaquine phosphate with chloroquine in acute, uncomplicated *Plasmodium vivax* malaria: a phase III, multicentric, open-label study. *Malar J* 2016; **15**: 42.

174. Bergonzoli G, Rivers Cuadra JC. [Therapeutic efficacy of different antimalarial regimens in the Costa Rica-Nicaragua border region]. *Rev Panam Salud Publica* 2000; **7**(6): 366-70.

175. Abdon NP, Pinto AY, das Silva Rdo S, de Souza JM. [Assessment of the response to reduced treatment schemes for vivax malaria]. *Rev Soc Bras Med Trop* 2001; **34**(4): 343-8.

176. Mohapatra MK, Padhiary KN, Mishra DP, Sethy G. Atypical manifestations of *Plasmodium vivax* malaria. *Indian J Malariol* 2002; **39**(1-2): 18-25.

177. da Silva Rdo S, Pinto AY, Calvosa VS, de Souza JM. [Short course schemes for vivax malaria treatment]. *Rev Soc Bras Med Trop* 2003; **36**(2): 235-9.

178. Nandy A, Addy M, Maji AK, Bandyopadhyay AK. Monitoring the chloroquine sensitivity of *Plasmodium vivax* from Calcutta and Orissa, India. *Ann Trop Med Parasitol* 2003; **97**(3): 215-20.

179. Ruebush TK, 2nd, Zegarra J, Cairo J, et al. Chloroquine-resistant *Plasmodium vivax* malaria in Peru. *Am J Trop Med Hyg* 2003; **69**(5): 548-52.

180. Kurcer MA, Simsek Z, Zeyrek FY, et al. Efficacy of chloroquine in the treatment of *Plasmodium vivax* malaria in Turkey. *Ann Trop Med Parasitol* 2004; **98**(5): 447-51.

181. Kurcer MA, Simsek Z, Kurcer Z. The decreasing efficacy of chloroquine in the treatment of *Plasmodium vivax* malaria, in Sanliurfa, south-eastern Turkey. *Ann Trop Med Parasitol* 2006; **100**(2): 109-13.

182. Walsh DS, Wilairatana P, Tang DB, et al. Randomized trial of 3-dose regimens of tafenoquine (WR238605) versus low-dose primaquine for preventing *Plasmodium vivax* malaria relapse. *Clin Infect Dis* 2004; **39**(8): 1095-103.

183. Dunne MW, Singh N, Shukla M, et al. A double-blind, randomized study of azithromycin compared to chloroquine for the treatment of *Plasmodium vivax* malaria in India. *Am J Trop Med Hyg* 2005; **73**(6): 1108-11.

184. Krudsood S, Wilairatana P, Tangpukdee N, et al. Safety and tolerability of elubaquine (bulaquine, CDRI 80/53) for treatment of *Plasmidium vivax* malaria in Thailand. *Korean J Parasitol* 2006; **44**(3): 221-8.

185. de Santana Filho FS, Arcanjo AR, Chehuan YM, et al. Chloroquine-resistant *Plasmodium vivax*, Brazilian Amazon. *Emerg Infect Dis* 2007; **13**(7): 1125-6.

186. Lee SW, Lee M, Lee DD, et al. Biological resistance of hydroxychloroquine for *Plasmodium vivax* malaria in the Republic of Korea. *Am J Trop Med Hyg* 2009; **81**(4): 600-4.

187. Liang GL, Sun XD, Wang J, Zhang ZX. [Sensitivity of *Plasmodium vivax* to chloroquine in Laza City, Myanmar]. *Zhongguo Ji Sheng Chong Xue Yu Ji Sheng Chong Bing Za Zhi* 2009; **27**(2): 175-6.

188. Rogers WO, Sem R, Tero T, et al. Failure of artesunate-mefloquine combination therapy for uncomplicated *Plasmodium falciparum* malaria in southern Cambodia. *Malar J* 2009; **8**: 10.

189. Leslie T, Rab MA, Ahmadzai H, et al. Compliance with 14-day primaquine therapy for radical cure of vivax malaria--a randomized placebo-controlled trial comparing unsupervised with supervised treatment. *Trans R Soc Trop Med Hyg* 2004; **98**(3): 168-73.

190. Alvarez G, Pineros JG, Tobon A, et al. Efficacy of three chloroquine-primaquine regimens for treatment of *Plasmodium vivax* malaria in Colombia. *Am J Trop Med Hyg* 2006; **75**(4): 605-9.

191. Carmona-Fonseca J, Uscategui RM, Correa AM. Vivax malaria in children: Clinical features and response to chloroquine. [Spanish]. *Colombia Medica* 2008; **39**(4): 364-77.

192. Orjuela-Sanchez P, da Silva NS, da Silva-Nunes M, Ferreira MU. Recurrent parasitemias and population dynamics of *Plasmodium vivax* polymorphisms in rural Amazonia. *Am J Trop Med Hyg* 2009; **81**(6): 961-8.

193. Daneshvar C, Davis TM, Cox-Singh J, et al. Clinical and parasitological response to oral chloroquine and primaquine in uncomplicated human *Plasmodium knowlesi* infections. *Malar J* 2010; **9**: 238.

194. Manandhar S, Bhusal CL, Ghimire U, Singh SP, Karmacharya DB, Dixit SM. A study on relapse/re-infection rate of *Plasmodium vivax* malaria and identification of the predominant genotypes of *P. vivax* in two endemic districts of Nepal. *Malar J* 2013; **12**: 324.

195. Shalini S, Chaudhuri S, Sutton PL, et al. Chloroquine efficacy studies confirm drug susceptibility of *Plasmodium vivax* in Chennai, India. *Malar J* 2014; **13**: 129.

196. Genton B, Baea K, Lorry K, Ginny M, Wines B, Alpers MP. Parasitological and clinical efficacy of standard treatment regimens against *Plasmodium falciparum*, *P. vivax* and *P. malariae* in Papua New Guinea. *P N G Med J* 2005; **48**(3-4): 141-50.

197. Kolaczinski K, Durrani N, Rahim S, Rowland M. Sulfadoxine-pyrimethamine plus artesunate compared with chloroquine for the treatment of vivax malaria in areas co-endemic for *Plasmodium falciparum* and *P. vivax*: a randomised non-inferiority trial in eastern Afghanistan. *Trans R Soc Trop Med Hyg* 2007; **101**(11): 1081-7.

198. Barnadas C, Ratsimbasoa A, Tichit M, et al. *Plasmodium vivax* resistance to chloroquine in Madagascar: clinical efficacy and polymorphisms in pvmdr1 and pvcrt-o genes. *Antimicrob Agents Chemother* 2008; **52**(12): 4233-40.

199. Teka H, Petros B, Yamuah L, et al. Chloroquine-resistant *Plasmodium vivax* malaria in Debre Zeit, Ethiopia. *Malar J* 2008; **7**: 220.

200. Ketema T, Getahun K, Bacha K. Therapeutic efficacy of chloroquine for treatment of *Plasmodium vivax* malaria cases in Halaba district, South Ethiopia. *Parasit Vectors* 2011; **4**: 46.

201. Anez A, Navarro-Costa D, Yucra O, et al. [Therapeutic response of *Plasmodium vivax* to chloroquine in Bolivia]. *Biomedica* 2012; **32**(4): 527-35.

202. Baird JK, Tiwari T, Martin GJ, et al. Chloroquine for the treatment of uncomplicated malaria in Guyana. *Ann Trop Med Parasitol* 2002; **96**(4): 339-48.

203. Pukrittayakamee S, Chantra A, Simpson JA, et al. Therapeutic responses to different antimalarial drugs in vivax malaria. *Antimicrob Agents Chemother* 2000; **44**(6): 1680-5.

204. Singh RK. Emergence of chloroquine-resistant vivax malaria in south Bihar (India). *Trans R Soc Trop Med Hyg* 2000; **94**(3): 327.

205. Villalobos-Salcedo JM, Tada MS, Kimura E, Menezes MJ, Pereira da Silva LH. In-vivo sensitivity of *Plasmodium vivax* isolates from Rond nia (western Amazon region, Brazil) to regimens including chloroquine and primaquine. *Ann Trop Med Parasitol* 2000; **94**(8): 749-58.

206. Buchachart K, Krudsood S, Singhasivanon P, et al. Effect of primaquine standard dose (15 mg/day for 14 days) in the treatment of vivax malaria patients in Thailand. *Southeast Asian J Trop Med Public Health* 2001; **32**(4): 720-6.

207. Dua VK, Sharma VP. Plasmodium vivax relapses after 5 days of primaquine treatment, in some industrial complexes of India. *Ann Trop Med Parasitol* 2001; **95**(7): 655-9.

208. Duarte EC, Pang LW, Ribeiro LC, Fontes CJ. Association of subtherapeutic dosages of a standard drug regimen with failures in preventing relapses of vivax malaria. *Am J Trop Med Hyg* 2001; **65**(5): 471-6.

209. Soto J, Toledo J, Gutierrez P, et al. *Plasmodium vivax* clinically resistant to chloroquine in Colombia. *Am J Trop Med Hyg* 2001; **65**(2): 90-3.

210. Maguire JD, Lacy MD, Sururi, et al. Chloroquine or sulfadoxine-pyrimethamine for the treatment of uncomplicated, *Plasmodium falciparum* malaria during an epidemic in Central Java, Indonesia. *Ann Trop Med Parasitol* 2002; **96**(7): 655-68.

211. Fryauff DJ, Leksana B, Masbar S, et al. The drug sensitivity and transmission dynamics of human malaria on Nias Island, North Sumatra, Indonesia. *Ann Trop Med Parasitol* 2002; **96**(5): 447-62.

212. Castillo CM, Osorio LE, Palma GI. Assessment of therapeutic response of *Plasmodium vivax* and *Plasmodium falciparum* to chloroquine in a Malaria transmission free area in Colombia. *Mem Inst Oswaldo Cruz* 2002; **97**(4): 559-62.

213. Congpuong K, Na-Bangchang K, Thimasarn K, Tasanor U, Wernsdorfer WH. Sensitivity of *Plasmodium vivax* to chloroquine in Sa Kaeo Province, Thailand. *Acta Trop* 2002; **83**(2): 117-21.

214. Hamedi Y, Nateghpour M, Tan-ariya P, Tiensuwan M, Silachamroon U, Looareesuwan S. *Plasmodium vivax* malaria in Southeast Iran in 1999-2001: establishing the response to chloroquine in vitro and in vivo. *Southeast Asian J Trop Med Public Health* 2002; **33**(3): 512-8.

215. Tjitra E, Baker J, Suprianto S, Cheng Q, Anstey NM. Therapeutic efficacies of artesunate-sulfadoxine-pyrimethamine and chloroquine-sulfadoxine-pyrimethamine in vivax malaria pilot studies: relationship to *Plasmodium vivax dhfr* mutations. *Antimicrob Agents Chemother* 2002; **46**(12): 3947-53.

216. Blair-Trujillo S, Castano AT, Restrepo ME, Sanchez GA, Carmona-Fonseca J. [Adequate clinical and parasitological *Plasmodium vivax* response to chloroquine in Colombia (Turbo, Antioquia), 2001]. *Infectio* 2002; **6**(1): 21-6.

217. Yadav RS, Ghosh SK. Radical curative efficacy of five-day regimen of primaquine for treatment of *Plasmodium vivax* malaria in India. *J Parasitol* 2002; **88**(5): 1042-4.

218. Machado RL, de Figuereido Filho AF, Calvosa VS, Figueredo MC, Nascimento JM, Povoa MM. Correlation between *Plasmodium vivax* variants in Belem, Para State, Brazil and symptoms and clearance of parasitaemia. *Braz J Infect Dis* 2003; **7**(3): 175-7.

219. Pinto AY, Azevedo CH, da Silva JB, de Souza JM. Assessment of chloroquine single dose treatment of malaria due to *Plasmodium vivax* in Brazilian Amazon. *Rev Inst Med Trop Sao Paulo* 2003; **45**(6): 327-31.

220. Rajgor DD, Gogtay NJ, Kadam VS, et al. Efficacy of a 14-day primaquine regimen in preventing relapses in patients with *Plasmodium vivax* malaria in Mumbai, India. *Trans R Soc Trop Med Hyg* 2003; **97**(4): 438-40.

221. Sumawinata IW, Bernadeta, Leksana B, et al. Very high risk of therapeutic failure with chloroquine for uncomplicated *Plasmodium falciparum* and *P. vivax* malaria in Indonesian Papua. *Am J Trop Med Hyg* 2003; **68**(4): 416-20.

222. Valibayov A, Abdullayev F, Mammadov S, et al. Clinical efficacy of chloroquine followed by primaquine for *Plasmodium vivax* treatment in Azerbaijan. *Acta Trop* 2003; **88**(1): 99-102.

223. Hapuarachchi HA, Dayanath MY, Abeysundara S, Bandara KB, Abeyewickreme W, de Silva NR. Chloroquine resistant falciparum malaria among security forces personnel in the Northern Province of Sri Lanka. *Ceylon Med J* 2004; **49**(2): 47-51.

224. Vijaykadga S, Rojanawatsirivej C, Congpoung K, et al. Assessment of therapeutic efficacy of chloroquine for vivax malaria in Thailand. *Southeast Asian J Trop Med Public Health* 2004; **35**(3): 566-9.

225. Maguire JD, Krisin, Marwoto H, Richie TL, Fryauff DJ, Baird JK. Mefloquine is highly efficacious against chloroquine-resistant *Plasmodium vivax* malaria and *Plasmodium falciparum* malaria in Papua, Indonesia. *Clin Infect Dis* 2006; **42**(8): 1067-72.

226. Tasanor O, Ruengweerayut R, Sirichaisinthop J, Congpuong K, Wernsdorfer WH, Na-Bangchang K. Clinical-parasitological response and in-vitro sensitivity of *Plasmodium vivax* to chloroquine and quinine on the western border of Thailand. *Trans R Soc Trop Med Hyg* 2006; **100**(5): 410-8.

227. Krudsood S, Tangpukdee N, Muangnoicharoen S, et al. Clinical efficacy of chloroquine versus artemether-lumefantrine for *Plasmodium vivax* treatment in Thailand. *Korean J Parasitol* 2007; **45**(2): 111-4.

228. Nateghpour M, Sayedzadeh SA, Edrissian GH, et al. Evaluation of Sensitivity of *Plasmodium vivax* to Chloroquine. *Iran J Public Health* 2007; **36**(3): 60-3.

229. Osorio L, Perez Ldel P, Gonzalez IJ. [Assessment of the efficacy of antimalarial drugs in Tarapaca, in the Colombian Amazon basin]. *Biomedica* 2007; **27**(1): 133-40.

230. Perez MA, Cortes LJ, Guerra AP, Knudson A, Usta C, Nicholls RS. [Efficacy of the amodiaquine+sulfadoxine-pyrimethamine combination and of chloroquine for the treatment of malaria in Cordoba, Colombia, 2006]. *Biomedica* 2008; **28**(1): 148-59.

231. Srivastava HC, Yadav RS, Joshi H, et al. Therapeutic responses of *Plasmodium vivax* and *P. falciparum* to chloroquine, in an area of western India where *P. vivax* predominates. *Ann Trop Med Parasitol* 2008; **102**(6): 471-80.

232. Nateghpour M, Edrissian G, Torabi A, et al. Monitoring of *Plasmodium vivax* and *Plasmodium falciparum* response to chloroquine in Bandar-Abbas district, Hormozgan province, Iran. [Arabic]. *Tehran Uni Med J* 2009; **67**(3): 178-83.

233. Dilmec F, Kurcer MA, Akkafa F, Simsek Z. Monitoring of failure of chloroquine treatment for *Plasmodium vivax* using polymerase chain reaction in Sanliurfa province, Turkey. *Parasitol Res* 2010; **106**(4): 783-8.

234. Kinzer MH, Chand K, Basri H, et al. Active case detection, treatment of falciparum malaria with combined chloroquine and sulphadoxine/pyrimethamine and vivax malaria with chloroquine and molecular markers of anti-malarial resistance in the Republic of Vanuatu. *Malar J* 2010; **9**: 89.

235. Takeuchi R, Lawpoolsri S, Imwong M, et al. Directly-observed therapy (DOT) for the radical 14-day primaquine treatment of *Plasmodium vivax* malaria on the Thai-Myanmar border. *Malar J* 2010; **9**: 308.

236. Yeshiwondim AK, Tekle AH, Dengela DO, Yohannes AM, Teklehaimanot A. Therapeutic efficacy of chloroquine and chloroquine plus primaquine for the treatment of *Plasmodium vivax* in Ethiopia. *Acta Trop* 2010; **113**(2): 105-13.

237. Asih PB, Syafruddin D, Leake J, et al. Phenotyping clinical resistance to chloroquine in *Plasmodium vivax* in northeastern Papua, Indonesia. *Int J Parasitol Drugs Drug Resist* 2011; **1**(1): 28-32.

238. Congpuon K, Satimai W, Sujariyakul A, et al. In vivo sensitivity monitoring of chloroquine for the treatment of uncomplicated vivax malaria in four bordered provinces of Thailand during 2009-2010. *J Vector Borne Dis* 2011; **48**(4): 190-6.

239. Maneeboonyang W, Lawpoolsri S, Puangsa-Art S, et al. Directly observed therapy with primaquine to reduce the recurrence rate of *Plasmodium vivax* infection along the Thai-Myanmar border. *Southeast Asian J Trop Med Public Health* 2011; **42**(1): 9-18.

240. Muhamad P, Ruengweerayut R, Chacharoenkul W, Rungsihirunrat K, Na-Bangchang K. Monitoring of clinical efficacy and in vitro sensitivity of *Plasmodium vivax* to chloroquine in area along Thai Myanmar border during 2009-2010. *Malar J* 2011; **10**: 44.

241. Van den Eede P, Soto-Calle VE, Delgado C, et al. *Plasmodium vivax* sub-patent infections after radical treatment are common in Peruvian patients: results of a 1-year prospective cohort study. *PLoS One* 2011; **6**(1): e16257.

242. Yohannes AM, Teklehaimanot A, Bergqvist Y, Ringwald P. Confirmed vivax resistance to chloroquine and effectiveness of artemether-lumefantrine for the treatment of vivax malaria in Ethiopia. *Am J Trop Med Hyg* 2011; **84**(1): 137-40.

243. Graf PC, Durand S, Alvarez Antonio C, et al. Failure of Supervised Chloroquine and Primaquine Regimen for the Treatment of *Plasmodium vivax* in the Peruvian Amazon. *Malar Res Treat* 2012; **2012**: 936067.

244. Pedro RS, Guaraldo L, Campos DP, Costa AP, Daniel-Ribeiro CT, Brasil P. *Plasmodium vivax* malaria relapses at a travel medicine centre in Rio de Janeiro, a non-endemic area in Brazil. *Malar J* 2012; **11**: 245.

245. Ganguly S, Saha P, Guha SK, et al. In vivo therapeutic efficacy of chloroquine alone or in combination with primaquine against vivax malaria in Kolkata, West Bengal, India, and polymorphism in *pvmdr1* and *pvcrt-o* genes. *Antimicrob Agents Chemother* 2013; **57**(3): 1246-51.

246. Leang R, Barrette A, Bouth DM, et al. Efficacy of dihydroartemisinin-piperaquine for treatment of uncomplicated *Plasmodium falciparum* and *Plasmodium vivax* in Cambodia, 2008 to 2010. *Antimicrob Agents Chemother* 2013; **57**(2): 818-26.

247. Liu H, Yang HL, Xu JW, Wang JZ, Nie RH, Li CF. Artemisinin-naphthoquine combination versus chloroquine-primaquine to treat vivax malaria: an open-label randomized and non-inferiority trial in Yunnan Province, China. *Malar J* 2013; **12**: 409.

248. Macareo L, Lwin KM, Cheah PY, Yuentrakul P, Miller RS, Nosten F. Triangular test design to evaluate tinidazole in the prevention of *Plasmodium vivax* relapse. *Malar J* 2013; **12**: 173.

249. Miahipour A, Keshavarz H, Heidari A, Raeisi A, Rezaeian M, Rezaei S. Assessment of the efficacy of 8 weeks of primaquine for the prevention of relapse in vivax malaria patients using SSCP-PCR and sequencing in South and South-East Iran, 2008-2011. *Trans R Soc Trop Med Hyg* 2013; **107**(7): 420-6.

250. Rios A, Alvarez G, Blair S. [Ten years of chloroquine efficacy for uncomplicated *Plasmodium vivax* malaria treatment, Turbo, Antioquia, 2002 and 2011]. *Biomedica* 2013; **33**(3): 429-38.

251. Zhu G, Lu F, Cao J, et al. Blood stage of *Plasmodium vivax* in central China is still susceptible to chloroquine plus primaquine combination therapy. *Am J Trop Med Hyg* 2013; **89**(1): 184-7.

252. Amaratunga C, Sreng S, Mao S, et al. Chloroquine remains effective for treating *Plasmodium vivax* malaria in Pursat province, Western Cambodia. *Antimicrob Agents Chemother* 2014; **58**(10): 6270-2.

253. Delgado-Ratto C, Soto-Calle VE, Van den Eede P, et al. Population structure and spatio-temporal transmission dynamics of *Plasmodium vivax* after radical cure treatment in a rural village of the Peruvian Amazon. *Malar J* 2014; **13**: 8.

254. Liu H, Yang HL, Tang LH, et al. Monitoring *Plasmodium vivax* chloroquine sensitivity along China-Myanmar border of Yunnan Province, China during 2008-2013. *Malar J* 2014; **13**: 364.

255. Rajgor DD, Gogtay NJ, Kadam VS, et al. Antirelapse Efficacy of Various Primaquine Regimens for *Plasmodium vivax*. *Malar Res Treat* 2014; **2014**: 347018.

256. Ould Ahmedou Salem MS, Mohamed Lemine YO, Deida JM, et al. Efficacy of chloroquine for the treatment of *Plasmodium vivax* in the Saharan zone in Mauritania. *Malar J* 2015; **14**: 39.

257. Assefa M, Eshetu T, Biruksew A. Therapeutic efficacy of chloroquine for the treatment of *Plasmodium vivax* malaria among outpatients at Hossana Health Care Centre, southern Ethiopia. *Malar J* 2015; **14**: 458.

258. Cheoymang A, Ruenweerayut R, Muhamad P, Rungsihirunrat K, Na-Bangchang K. Patients' adherence and clinical effectiveness of a 14-day course of primaquine when given with a 3-day chloroquine in patients with *Plasmodium vivax* at the Thai-Myanmar border. *Acta Trop* 2015; **152**: 151-6.

259. Pareek A, Chandurkar N, Gogtay N, et al. Sustained Release Formulation of Primaquine for Prevention of Relapse of *Plasmodium vivax* Malaria: A Randomized, Double-Blind, Comparative, Multicentric Study. *Malar Res Treat* 2015; **2015**: 579864.

260. Yuan L, Wang Y, Parker DM, et al. Therapeutic responses of *Plasmodium vivax* malaria to chloroquine and primaquine treatment in northeastern Myanmar. *Antimicrob Agents Chemother* 2015; **59**(2): 1230-5.

261. Beyene HB, Beyene MB, Ebstie YA, Desalegn Z. Efficacy of Chloroquine for the Treatment of Vivax malaria in Northwest Ethiopia. *PLoS One* 2016; **11**(8): e0161483.

262. Lo E, Nguyen J, Oo W, et al. Examining *Plasmodium falciparum* and *P. vivax* clearance subsequent to antimalarial drug treatment in the Myanmar-China border area based on quantitative real-time polymerase chain reaction. *BMC Infect Dis* 2016; **16**: 154.

263. Longley RJ, Sripoorote P, Chobson P, et al. High Efficacy of Primaquine Treatment for *Plasmodium vivax* in Western Thailand. *Am J Trop Med Hyg* 2016; **95**(5): 1086-9.

264. Negreiros S, Farias S, Viana GM, et al. Efficacy of Chloroquine and Primaquine for the Treatment of Uncomplicated *Plasmodium vivax* Malaria in Cruzeiro do Sul, Brazil. *Am J Trop Med Hyg* 2016; **95**(5): 1061-8.

265. Waqar T, Khushdil A, Haque K. Efficacy of Chloroquine as a first line agent in the treatment of uncomplicated malaria due to *Plasmodium vivax* in children and treatment practices in Pakistan: A Pilot study. *J Pak Med Assoc* 2016; **66**(1): 30-3.

266. Seifu S, Zeynudin A, Zemene E, Suleman S, Biruksew A. Therapeutic efficacy of chloroquine for the treatment of *Plasmodium vivax* malaria among outpatients at Shawa Robit Health Care Centre, North-East Ethiopia. *Acta Trop* 2017; **171**: 44-51.
